# A Novel Expert System for Diagnosis of Iron Deficiency Anemia

**DOI:** 10.1155/2022/7352096

**Published:** 2022-10-14

**Authors:** Erol Terzi, Bünyamin Sarıbacak, Fatih Sağlam, Mehmet Ali Cengiz

**Affiliations:** Department of Statistics, Faculty of Art and Sciences, Ondokuz Mayis University, Samsun, Turkey

## Abstract

Diagnosis of a disease is one of the most important processes in the field of medicine. Thus, computer-aided detection systems are becoming increasingly important to assist physicians. The iron deficiency anemia (IDA) is a serious health problem that requires careful diagnosis. Diagnosis of IDA is a classification problem, and there are various studies conducted. Researchers also use feature selection approaches to detect significant variables. Studies so far investigate different classification problems such as outliers, class imbalance, presence of noise, and multicollinearity. However, datasets are usually affected by more than one of these problems. In this study, we aimed to create multiple systems that can separate diseased and healthy individuals and detect the variables that have a significant effect on these diseases considering influential classification problems. For this, we prepared different datasets based on the original dataset whose outliers were removed using different outlier detection methods. Then, a multistep classification algorithm was proposed for each dataset to see the results under irregular and regulated conditions. In each step, a different classification problem is handled. The results showed that it is important to consider each question together as it can and should change the outcome. Dataset and *R* codes used in the study are available as supplementary files online.

## 1. Introduction

Iron is a component of every living cell and is an essential element for maintaining health. Iron participates in a large number of biochemical reactions, mainly related to oxygen transport and storage, the production of adenosine triphosphate, the synthesis of deoxyribonucleic acid, and electron transport [1]. Iron metabolism is controlled by absorption rather than excretion. Iron is lost only through blood loss or cell loss as it is shed. Men and women who do not have menstruation lose about 1 mg of iron per day. Iron deficiency occurs when the body's iron needs are not met by iron absorption from the diet [2]. Anemia is a condition in which hemoglobin is less than normal, and the oxygen carrying capacity of the blood is reduced to meet the physiological needs of the body [3]. Approximately 43% of children under the age of 5 and 29% of nonpregnant women of reproductive age worldwide are anemic [4]. The prevalence varies significantly between countries and is especially high in India [5]. Iron (Fe) deficiency has been recognized as the most common cause of anemia and is associated with about 25-50% of anemia worldwide [6–8].

There are previous studies in this field in the literature. Yildiz et al. [9] propose a system to enable the recognition of anemia in general clinical practice conditions. For this system, a model was created using four different artificial learning methods. Artificial neural networks, support vector machines, Naive Bayes, and ensemble decision tree methods were used as classification algorithms, and they achieved the highest accuracy rate with the bagged decision tree method (85.60%). The differential diagnosis of IDA and *β*-thalassemia was made by using machine learning techniques such as RBC indices, support vector machine (SVM), and *K*-nearest neighbor (KNN) by Ayyıldız and Tuncer [10]. Çil et al. [11] developed a decision support system to distinguish between *β*-thalassemia and IDA using logistic regression, *K*-nearest neighbors, support vector machine, extreme learning machine, and regularized extreme learning machine classification algorithms. Nur et al. [12] investigated the IDA in children with severe caries undergoing dental surgery under general anesthesia. Azarkhish et al. [13] developed an artificial neural network (ANN) and an adaptive neurofuzzy inference system (ANFIS) to diagnose the IDA and predict serum iron levels. Yılmaz and Bozkurt [14] developed an application by using different machine learning algorithms to diagnose IDA in women. They achieved 97.60% sensitivity and 99.16% accuracy using feed forward distributed time delay. Yılmaz et al. [15] introduced a fuzzy expert system which determines the level of IDA. Dogan and Turkoglu [16] proposed a decision tree system to detect IDA from hematology. Yavuz et al. [17] used decision tree based on feed forward network and KNN to diagnose IDA.

The main purpose of this study is to create a system that can diagnose IDA in an individual in a computer environment using real data samples belonging to individuals and identify variables that are important for IDA diagnosis in different situations. Under this purpose, a system that addresses each of the outlier, noise, class imbalance, and variable selection problems encountered in the dataset belonging to real patients has been proposed. The proposed system achieves the variable effects and performance scores observed in the presence of outliers separately along with the variable effects and performances observed in the absence of outliers. In this system, the original data can be examined both without discarding outliers and by creating a total of four different models in which outliers are discarded using three different methods.

We used *Z*-score, relative density-based outlier factor (RDOS) [18], and natural outlier factor (NOF) [19] as outlier detection. The *Z*-score method is preferred because it is the most basic method, while RDOS and NOF are some of the most recent outlier detection methods. Then, we selected the important variables with the method Boruta feature selection [20].

Oversampling and undersampling methods are used to eliminate class imbalance. The synthetic minority oversampling technique (SMOTE) [21] is preferred as the oversampling method since it is the most well-known resampling method. SMOTE is a technique that is vulnerable to noise in data [22–26]. Thus, the ensemble filter (EF) noise detection method [27] is used as the noise detection and undersampling method to establish models resistant to the presence of noise. The reason why this method is preferred is that it has performed successfully together with SMOTE before [28]. Extreme gradient boosting (XGBoost) used by Chen and Guesthin [29] was preferred as a classification method since it has proven to be a successful method in many studies such as Sandulescu and Chiru [30], Abel et al. [31], Anelli et al. [32], and Cogranne et al. [33]. The XGBoost method is a method that can make the variable selection in itself. In this way, model-based variable selection is also made within crossvalidation. In addition, XGBoost is not affected by multicollinearity [34, 35]. Thus, we made it possible to establish classification models that are resistant to the existence of all the problems mentioned. What distinguishes this study from other studies is that it can address the most important classification problems altogether.

The paper consists of four sections. The first section, the introduction, gives a summary of IDA and literature on the problem. The second section explains the dataset, proposed system, and its flowchart and details about methods used in the system. The third section gives the results belonging to the dataset. The fourth and last section gives conclusive interoperations.

## 2. Materials and Method

### 2.1. Collecting Data

Between October 2017 and March 2020, 516 cases diagnosed with malaise and fatigue (ICD-10 code: R53) who applied to the Samsun Training and Research Hospital, Hematology Department were retrospectively analyzed. IDA was diagnosed in 359 patients by looking at laboratory results. The remaining 157 cases were evaluated with the same diagnosis, and their laboratory values were not compatible with IDA: age, gender, hemoglobin (Hb), hematocrit (Hct), mean corpuscular volume (MCV), mean corpuscular hemoglobin concentration (MCHC), red cell distribution width (RDW), red blood cell (RBC) and IDA parameters, iron (Fe), ferritin (FERR), unsaturated iron binding capacity (UIBC). There are 516 cases were recorded. The abbreviations are shown in Table 1. DD is the response variable. It has two classes, true and false, true means the person has IDA, and false if vice versa. The data set is included within the supplementary information file (available here).

### 2.2. Proposed Classification System

**Figure 1:**
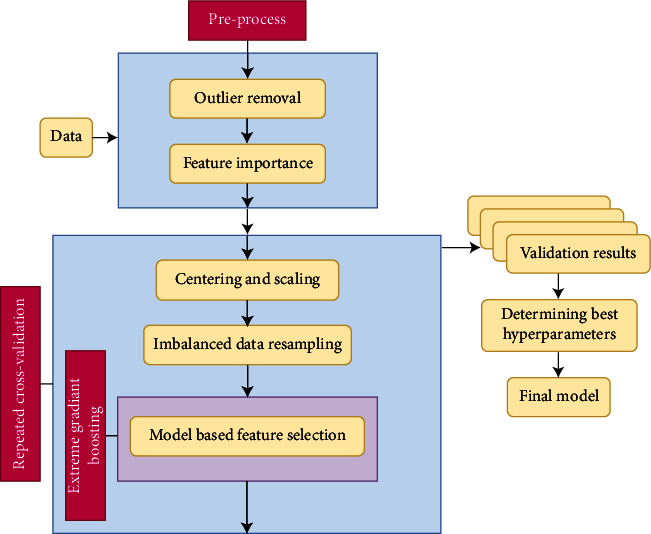
Flowchart of proposed system.

We have two goals in this study. The first is to obtain a model that can determine whether an individual has IDA or not, is resistant to the problem of class imbalance and noise, and makes a choice of variables within itself. Secondly, to determine the effectiveness of variables and the parameters of the best model in cases of the presence and absence of outliers.  Figure 1 gives the flowchart of the proposed system. Before crossvalidation, outliers are detected and removed. Outlier detection methods are *Z*-score, RDOS, and NOF. The variable selection process has two stages, before and during crossvalidation. Whether the variables belonging to these data sets are significant before crossvalidation is determined by Boruta. In the crossvalidation, the training set is transformed to have zero mean and one variance. The class imbalance and noise problems are solved by oversampling using SMOTE and noise detection-undersampling EF.

XGBoost is used to form classification models. The hyperparameters belonging to XGBoost have been determined to maximize Matthew's correlation coefficient performance with grid search because class imbalance has been taken into account. In this way, this system will establish the most appropriate model that can make an IDA diagnosis suitable for every situation in a way that takes into account each of the major classification problems.

### 2.3. Outlier Removal

Outlier is defined by Hawkings [36] as “An observation which deviates so much from other observations as to arouse suspicions that it was generated by a different mechanism.”

Outliers may cause the model to make incorrect predictions, incorrect feature selection, and misleading performance measurements.

#### 2.3.1. *Z*-Score

The *Z*-score method is a univariate outlier detection method. In this method, *Z*-scores are obtained by standardizing the values of each variable. By taking the absolute value of *Z*-scores, samples above a certain threshold value are considered outliers. After removing the outlier, the process is repeated until a certain number of outliers are detected or a new sample cannot be found on the remaining samples.

#### 2.3.2. Relative Density-Based Outlier Factor (RDOS)

Given a set of objects *X* = {*X*_1_, *X*_2_, ⋯, *X*_*n*_}, where *X*_*i*_ ∈ ℝ^*d*^ for *i* = 1, ⋯, *n* kernel density, estimation (KDE) estimates the distribution as

(1) $$\begin{array}{c} p\left(X\right)=\frac{1}{n}\overset{n}{\underset{i=1}{\sum }}\frac{1}{h^{d}}K\left(\frac{X-X_{i}}{h}\right), \end{array}$$

where *K*(*X* − *X*_*i*_/*h*) is the defined kernel function with the kernel width of *h*. To estimate the density at the location of the object *X*_*j*_, only its neighbors of *X*_*j*_ as kernels considered. To better estimate the density distribution in the neighborhood of an object, *k* nearest neighbors, reverse nearest neighbors, and shared nearest neighbors are used. Let *NN*_*r*_(*X*_*j*_) be the *r*-th nearest neighbors of *X*_*j*_. *k* nearest neighbors of *X*_*j*_ as *S*_*KNN*_(*X*_*j*_) is denoted as

(2) $$\begin{array}{c} S_{\text{KNN}}\left(X_{j}\right)=\left\{\text{N}{\text{N}}_{1}\left(X_{j}\right),\text{N}{\text{N}}_{2}\left(X_{j}\right),\cdots ,\text{N}{\text{N}}_{k}\left(X_{j}\right)\right\}. \end{array}$$

The reverse nearest neighbors of *X*_*j*_ are those who consider *X*_*j*_ as one of their *k* nearest neighbors. The shared nearest neighbors are those who share one or more nearest neighbors with *X*_*j*_.

Let *S*_RNN_(*X*_*j*_) and *S*_SNN_(*X*_*j*_) be reverse nearest neighbors and shared nearest neighbors of *X*_*j*_, respectively. An extended local neighborhood, *S*(*X*_*j*_), is obtained by combination of three datasets, *S*_KNN_(*X*_*j*_) ∪ *S*_RNN_(*X*_*j*_) ∪ *S*_SNN_(*X*_*j*_). Estimated density at *X*_*j*_ is written as

(3) $$\begin{array}{c} p\left(X_{j}\right)=\frac{1}{\left|S\left(X_{j}\right)+1\right|}\underset{X\in S\left(X_{j}\right)\cup \left\{X_{j}\right\}}{\sum }\frac{1}{h^{d}}\left(\frac{X-X_{j}}{h}\right), \end{array}$$

where |*S*| denotes the number of elements in the set of *S*. Relative density-based outlier factor (RDOS) is then obtained by

(4) $$\begin{array}{c} \text{RDO}{\text{S}}_{k}\left(X_{j}\right)=\frac{\sum_{X_{i}\in S\left(X_{j}\right)}p\left(X_{i}\right)}{\left|S\left(X_{j}\right)\right|p\left(X_{j}\right)}. \end{array}$$

RDOS is the ratio of the average neighborhood density to the density of *X*_*j*_. Higher RDOS_*k*_(*X*_*j*_) value means higher outlierness of *X*_*j*_.

#### 2.3.3. Natural Outlier Factor (NOF)

If an object *X*_*j*_ considers *X*_*l*_ to be a neighbor and *X*_*l*_ considers *X*_*j*_ to be a neighbor at the same time, then *X*_*l*_ is called a natural neighbor of *X*_*j*_. Algorithm of natural neighbor searching (NaN-Searching) is given in Algorithm 1.

Rnb(*i*) is the times that point *i* contained by the neighborhood of other points, which the number of *i*'s reverse neighbor. sup_*k*_ is called natural eigenvalue and is the average value of the number of each point's neighbors. maxRnb(*i*) is called natural value.

The point which Rnb(*i*) = 0 after Algorithm 1 is natural outlier. The Natural Influence Space (NIS) is defined as

(5) $$\begin{array}{c} \text{NIS}\left(X_{j}\right)=\text{N}{\text{N}}_{k}\left(X_{j}\right)⨆\text{RN}{\text{N}}_{k}\left(X_{j}\right). \end{array}$$

The Natural Outlier Factor (NOF) is defined as

(6) $$\begin{array}{c} \text{NOF}\left(X_{j}\right)=\frac{\sum_{q\in NIS\left(X_{l}\right)}Ird_{k}\left(X_{l}\right)}{\left|\text{NIS}\left(X_{j}\right)\right|Ird_{k}\left(X_{j}\right)}. \end{array}$$

Here, *Ird*_*k*_(*X*_*j*_) is local reachability density and defined as

(7) $$\begin{array}{c} \text{Ir}{\text{d}}_{k}\left(X_{j}\right)=\frac{\left|NN_{k}\left(X_{j}\right)\right|}{\Sigma_{X_{m}\in NN_{k}\left(X_{j}\right)reach-dist_{k}\left(X_{j},X_{m}\right)}},\\ \text{reach}-\text{dis}{\text{t}}_{k}\left(X_{j},X_{m}\right)=\max\left\{k-\text{dist}\left(X_{m}\right),d\left(X_{j},X_{m}\right)\right\}. \end{array}$$

NOF(*X*_*j*_) gives the degree of outlierness of *X*_*j*_. Higher NOF(*X*_*j*_) value means higher outlierness of *X*_*j*_.

### 2.4. Boruta Feature Selection

Boruta is a method that decides whether the effect of the features is statistically significant using the random forest algorithm. It creates shadow features with randomness and determines the significance of the effects of the features by referencing these features. The randomness created in shadow features will reduce the relationship of these feature with the response. The Boruta process works as follows. Duplicate features of all features are included in the system. No matter how many features are in the system, at least 5 copies are added. By mixing these randomly within themselves, the relationship with the response is removed. A random forest model is created for each run on the new system, and feature importance levels are calculated. The shadow feature with maximum feature importance is called shadowMax. Feature with higher feature importance than shadowMax is significant. Hypothesis tests are used to determine significant features. The feature that has statistically higher importance than shadowMax is significant. If it has lower importance than shadowMax, then it is insignificant and can be removed. If there are statistically significant difference, then it is undetermined. Boruta continues until the specified number of runs or until statistically significant results are obtained for all features.

### 2.5. Imbalanced Data Resampling

Classification is a kind of pattern recognition method and assigns each input value to a value in the vector of “classes.” In almost all of the real data, the numbers of observations pertaining to classes show a skewed distribution, which, then is called class imbalance. Resampling methods are preprocessing methods in which the dataset is modified so that the classes of observations in the dataset become more balanced [37]. Past studies have shown that modeling by achieving class balance with resampling methods is a useful solution approach [38–40]. Resampling is divided into three categories as undersampling, oversampling, and mixed. Undersampling is to reach class balance by reducing majority class instances. Oversampling, on the other hand, aims to provide class balance by increasing the instances of minority classes. Mixed methods use undersampling and oversampling methods together.

#### 2.5.1. Ensemble Filter (EF)

Noise filtering methods are popular undersampling methods for imbalanced data sets. EF is a noise detection method which uses *m* weak base-level classifiers. Dataset is partitioned into train and test datasets. All instances are used as test set data *m* number of times. If an instance is wrongly predicted by all *m* classifiers, then it is tagged as noise.

#### 2.5.2. Synthetic Minority Oversampling Technique (SMOTE)

The SMOTE algorithm generates artificial data between existing positive observations based on the gaps in the feature space [41]. For *X*^pos^ ∈ *X* subset and *x*_*i*_ ∈ *X*^pos^, consider the nearest *k* neighbors of *x*_*i*_. Using the Euclidean distances between *x*_*i*_ and nearest neighbors ${\hat{x}}_{i}$ and a *λ* random uniformly distributed value between [0, 1], new synthetic sample is created as

(8) $$\begin{array}{c} x^{syn}=x_{i}+\left({\hat{x}}_{i}-x_{i}\right)\times \lambda . \end{array}$$

The synthetic sample, *x*^*syn*^, created from this equation becomes a random point on the line between *x*_*i*_ and its nearest neighbor ${\hat{x}}_{i}$. A pseudocode for the SMOTE algorithm used in this study is given in Algorithm 2. Here, *n*^syn^, *n*^pos^, and *n*^neg^ are the number of points in synthetic, positive class, and negative class datasets, respectively.

### 2.6. eXtreme Gradient Boosting (XGBoost)

XGBoost is one of the implementations of gradient boosting machines (gbm) which is known as one of the best performing algorithms utilized for supervised learning. The way the XGBoost works is as follows: if we have for example a dataset *D* that has *p* features and *n* number of examples *D* = {(*x*_*i*_, *y*_*i*_): *i* = 1, ⋯, *n*, *x*_*i*_ ∈ ℝ^*p*^, *y*_*i*_ ∈ ℝ}. Let ${\hat{y}}_{i}$ be the predicted output of an ensemble tree model generated from the following equations:

(9) $$\begin{array}{c} A_{i}=\phi \left(x_{i}\right)=\overset{K}{\underset{k=1}{\sum }}f_{k}\left(x_{i}\right),f_{k}\in F, \end{array}$$

where *K* represents the number of trees in the model, *f*_*k*_ represents the *k*-the tree, to solve the above equation, and we need to find the best set of functions by minimizing the loss and regularization objective.

(10) $$\begin{array}{c} L\left(\phi \right)=\underset{i}{\sum }l\left(y_{i},A_{i}\right)+\underset{k}{\sum }\Omega \left(f_{k}\right), \end{array}$$

where *l* represents the loss function which is the difference between the predicted output ${\hat{y}}_{i}$ and the actual output *y*_*i*_. While *Ω* is a measure of how complex the model is, this assists in avoiding overfitting of the model, and it is calculated using

(11) $$\begin{array}{c} \Omega \left(f_{k}\right)=\gamma T+\frac{1}{2}\lambda {\left\|w\right\|}^{2}. \end{array}$$

*T*, in the above equation, represents the number of leaves of the tree, and *w* is the weight of each leaf.

Decision trees to minimize the objective function boosting are used in the training the model, which works by adding a new function *f* as the model keeps training. So, in the *t*-th iteration, a new function (tree) is added as follows:

(12) $$\begin{array}{c} L^{\left(t\right)}=\overset{n}{\underset{i=1}{\sum }}l\left(y_{i},A_{i}^{\left(t-1\right)}+f_{t}\left(x_{i}\right)\right)+\Omega \left(f_{t}\right),\\ L_{\text{split}}=\frac{1}{2}\left[\frac{{\left(\sum_{i\in l_{L}}g_{i}\right)}^{2}}{\sum_{i\in l_{L}}h_{i}+\lambda }+\frac{{\left(\sum_{i\in l_{R}}g_{i}\right)}^{2}}{\sum_{i\in l_{R}}h_{i}+\lambda }-\frac{{\left(\sum_{i\in l}g_{i}\right)}^{2}}{\sum_{i\in l}h_{i}+\lambda }\right]-\gamma ,\\ g_{i}=\partial_{A^{\left(t-1\right)}}l\left(y_{i},A^{\left(t-1\right)}\right),\\ h_{i}=\partial_{A^{\left(t-1\right)}}^{2}l\left(y_{i},A^{\left(t-1\right)}\right). \end{array}$$

XGBoost speeds up the tree construction and uses a different algorithm for tree searching than earlier gradient boosting methods [42]. Compared to earlier gradient boosting algorithms, XGBoost is computationally efficient [43]. Because of its high speed out of core computation, data scientists prefer to use it commonly [44]. It also has shown satisfactory results in machine learning competitions [45].

### 2.7. Performance Metrics

Performance evaluations of the models can be calculated using confusion matrix and ROC curves.  Table 2 gives confusion matrix table. Here, TP is true-positive, FN is false-negative, FP is false-positive, and TN is true-negative predictions. Metrics accuracy (ACC), Matthew's correlation coefficient (MCC), sensitivity (Sens), and specifity (Spec) can be calculated using Table 2 as follows:

(13) $$\begin{array}{c} \text{ACC}=\frac{\text{TP}+\text{TN}}{\text{TP}+\text{TN}+\text{FP}+\text{FN}},\\ \text{MCC}=\frac{\text{TP}\times \text{TN}-\text{FP}\times \text{FN}}{\sqrt{\left(\text{TP}+\text{FP}\right)\left(\text{TP}+\text{FN}\right)\left(\text{TN}+\text{FP}\right)\left(\text{TN}+\text{FN}\right)}},\\ \text{Sens}=\frac{\text{TP}}{\text{TP}+\text{FN}},\\ \text{Spec}=\frac{\text{TN}}{\text{TN}+\text{FP}}. \end{array}$$

Area under curve (AUC) is calculated by calculating the area under ROC curve.

Here, the positive class means the minority class, and the negative class means the majority class. When it comes to class imbalance, all mentioned metrics will have bias towards the negative class. When we examine the literature, we see that all these criteria have different usage areas. Despite this, many studies say that the MCC criterion is more advantageous than other criteria [46–48].

## 3. Results

For outlier detection, we used *Z*-score, RDOS, and NOF methods. For *Z*-score, we selected threshold as 3. For RODS and NOF, outlier scores are investigated using *Z*-scores of theirs. Again, threshold is selected as 3. As a result, we obtained 4 different datasets which are summarized in Tables 3 and 4. In Table 3, if we consider the average ranges for a person, we see considerable extreme values in the maximum values of MCHC, RDW, Fe, UIBC, and FERR values. Also, there are obvious extreme values in the minimum values of Fe (1 mcg/dL), FERR (1 ng/mL), and UIBC (4.78 mcg/dL). Since there is always the possibility of mismeasuring or misrecording, we used outlier detection methods and obtained new ranges for these variables. Even after removing outliers, minimum values of Fe an FERR still shows extreme values. Also, maximum values of FERR of RDOS and NOF and RDW of NOF are still extremely high. When we look at Table 4, we see that the method that finds the highest number of outliers is RDOS. However, *Z*-score removed more false outliers.

**Figure 2:**
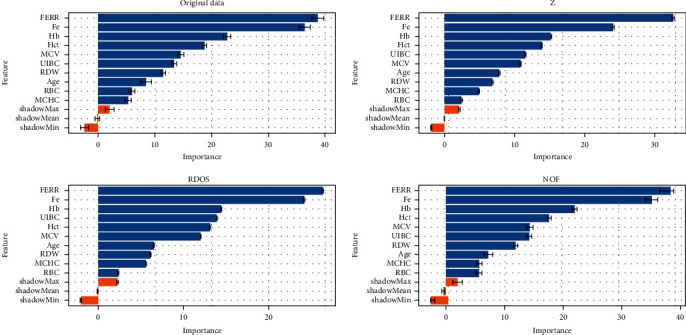
Boruta feature importance of each dataset.

Figure 2 gives the feature importance levels for the Boruta feature selection method. According to Boruta, all features have significant effect on response. There are comparative differences in original data and datasets which outliers are removed. FERR seems to be more important after when data is clean. RBC and MCHC are among the less important ones in all four datasets. In RDOS and *Z*, RBC is almost redundant. As conclusion, we can use all features in crossvalidation for all datasets.

**Figure 3:**
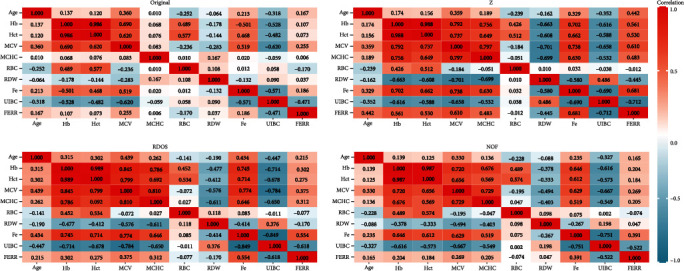
Correlation matrixes for four datasets.

Figure 3 shows the correlation matrixes of four datasets. It seems that Hb and Hct have a correlation of almost 1. There are various absolute correlations above 0.7. MCV seems to have increased correlation with other features after outliers are removed. There seems to be high multicollinearity in original dataset but higher multicollinearity in datasets without outliers.

**Figure 4:**
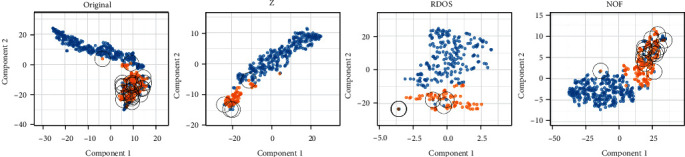
Scatter plots of each dataset for two component tsne.

Figure 4 shows the scatter plots of first two component of *t*-Distributed Stochastic Neighbour Embedding (tsne). Using tsne, we can see how classes are separated in a multivariate space and how they are not. Original data and NOF seem to have overlapped class instances which are noise. *Z* and RDOS datasets seem to have no problem about noise.

Now, we have four datasets, as each have different and similar problems. Original dataset and NOF have noise and multicollinearity problem. *Z* and RDOS dataset have class imbalance and multicollinearity problem. Since XGBoost is a method that is robust to multicollinearity, it should select only the most informative of the correlated features.

Repeated crossvalidation is conducted to determine the best hyper parameters and performances of each model. In order to tune hyperparameters of XGBoost models, we used grid search.  Table 5 gives the hyperparameters, grid search values, and best hyperparameters for each model. We formed models using *R* package “xgboost” [44]. We used available four of the available hyperparameters to tune. nrounds is the max number of boosting iterations, lambda is L2 regularization term on weights, alpha is L1 regularization term on weights, and eta is the learning rate. Best hyperparameters are determined according to MCC value of crossvalidation.

**Figure 5:**
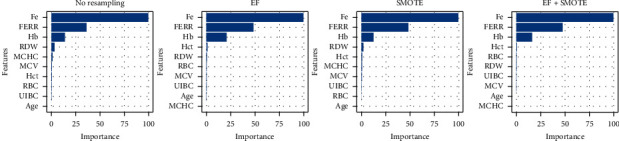
Original data XGBoost feature importance.

**Figure 6:**
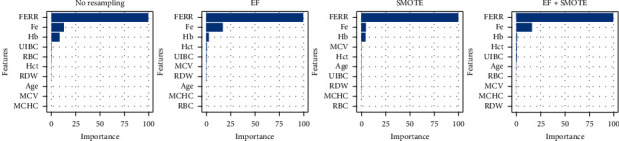
*Z* data XGBoost feature importances.

**Figure 7:**
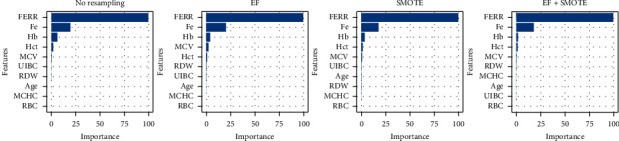
RDOS data XGBoost feature importance.

**Figure 8:**
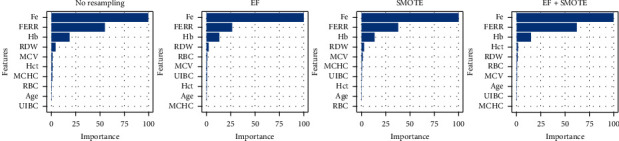
NOF data XGBoost feature importance.

Figures 5–8 give the average feature importance of XGBoost models for original, *Z*, DFOS, and NOF datasets, respectively. In original dataset, Fe, FERR, and Hb features are used, and other features have almost nonexistent effect. The most important feature is Fe for original dataset. For datasets cleaned by *Z* score and DFOS, we see the effect of FERR more clearly compared to Fe. For EF + SMOTE models, even the Hb's effect is redundant. For dataset cleaned by NOF, results are similar to original dataset. Fe is the most informative feature, and FERR and Hb are other features which are informative to diagnose IDA.

Tables 6–9 give performance results for original, *Z*, RDOS, and NOF datasets, respectively. Original dataset had outlier, noise, and multicollinearity problems.  Table 7 shows that best ACC, MCC, and Spec values are obtained for the EF + SMOTE method. For AUC and Sens, no resampling was needed. Since our negative (majority) class is true, which means the individual has IDA, without resampling, we expect the model to be biased towards true. As we can see in Table 8, sensitivity of no resampling model is the highest among them. If we look at a more balanced measure like MCC, the best model is the EF + SMOTE model.

Dataset cleaned based on *Z*-scores had the most imbalance rate of the four datasets. It did not have a noise problem compared to original and NOF dataset. Therefore, we expect EF to be not that effective. When we look at the performances, we see that SMOTE is the most successful one for ACC, MCC, AUC, and Sens. EF + SMOTE achieves for the best value for Spec. RDOS dataset had less imbalance and noise problem. Probably, the cleanest dataset is RDOS dataset. Performances in Table 8 show us that the best model for ACC, MCC, and sensitivity is achieved without resampling. Also, SMOTE gives the best AUC, and EF + SMOTE gives the best Spec value.

NOF dataset is the most similar to the original dataset. It had noise and multicollinearity problem.  Table 9 shows that the best score for AUC is achieved without resampling. EF + SMOTE achieves the best ACC, MCC, and Spec values. Also, the best Sens is achieved by the EF model.

**Figure 9:**
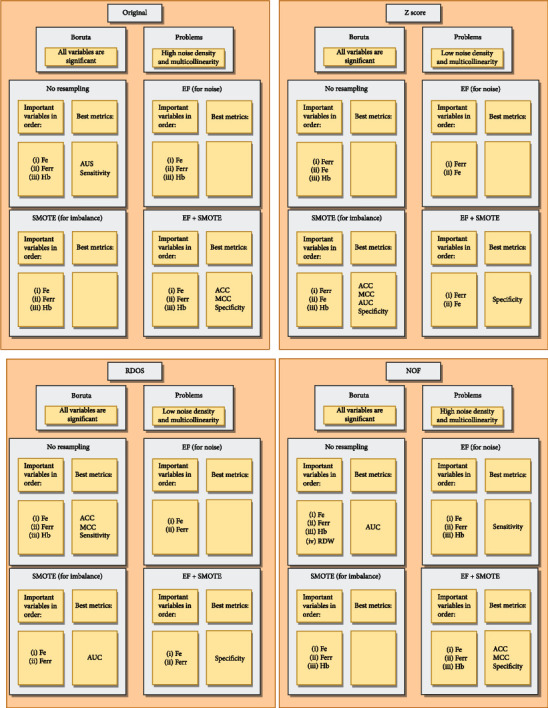
Overall results for four datasets.

Figure 9 gives overall results for four datasets together with problems and methods.

## 4. Discussion

The RDOS method was the one that found the most outliers, and the NOF method was the one that found the least outliers. The *Z*-score selected the outliers more from healthy individuals, while the RDOS and NOF method selected them more from IDA patients.

Boruta determined that the effects of all variables are significant in the original dataset before crossvalidation. High noise density was observed in the tsne graphs of the data set in which important variables were used. When we looked at the correlations between the variables, we realized that there is multicollinearity. We used XGBoost to model in order not to be affected by the multicollinearity. When SMOTE is applied to solve the class imbalance, and EF is applied to solve the noise problem. We have seen that the combined application is successful in ACC, MCC, and specificity metrics. This showed us that in situations where noise and class imbalance problems exist, approaches that consider both, rather than just one problem, are successful. The XGBoost method found Fe, Ferr, and Hb variables to be important for all cases, in order of importance. Variable importance did not differ by method.

We found that all variables were important before crossvalidation in the data set where outliers were eliminated with the *Z*-score. tsne graphs showed little noise density. Problems of multicollinearity and class imbalance still exist. When we did not address the noise problem but the class imbalance problem, the best models were obtained in ACC, MCC, AUC, and sensitivity metrics. It was enough to apply only SMOTE. The variable importance levels of XGBoost differed from the original dataset. For the models of the *Z*-score dataset, the Ferr variable is more important than the Fe variable in the absence of outliers. No effect of Hb variable was observed in EF and EF + SMOTE methods.

All variables are important when eliminating outliers with RDOS. As in the *Z*-score, the noise density is low, and class imbalance and multicollinearity are present. In this case, the best performance was achieved in the ACC, MCC, and sensitivity metrics when no resampling method was used. The SMOTE model achieved the best performance in AUC, and the EF + SMOTE model in specificity metrics. The EF model failed compared to other models. We have seen that class imbalance does not pose a problem for the performance of the model in terms of ACC, MCC, and sensitivity in this dataset, which already has a low noise ratio. XGBoost found variable importance and order of importance in the RDOS dataset, similar to the original dataset, but he found the variable Hb meaningless for EF, SMOTE, and EF + SMOTE.

When we discarded outliers with NOF, there was no reduction in noise density compared to other outlier methods. The dataset includes noise, class imbalance ,and multicollinearity problems. Boruta found all variables significant. The EF + SMOTE model was successful on most metrics (ACC, MCC, and specificity) as it addresses both noise and class imbalance problem. While XGBoost found Fe, Ferr, and Hb variables to be important in all models in order of importance, it achieved the best AUC performance when resampling was not applied and found the RDW variable to be important.

For all datasets, cases where all the problems were handled at once, instead of examining the problems one by one, allowed us to obtain better results, but there are some points to be noted: First is the correct selection of the outlier method. This study does not make a recommendation about which method should be used to detect outliers. In addition, the problems must be well identified. We have shown that successful results can be obtained when the presence of noise and class balance is correctly detected. However, different methods should be explored for noise detection and class imbalance resampling. The interpretations of each of the performance measures also vary and is used for different circumstances. Once the researcher has identified all of them correctly, he should tackle all the problems together, as we suggest.

## 5. Conclusion

This study is aimed at modeling IDA results of patients using auxiliary variables considering different classification problems. We have argued that dealing with all the problems together will lead to more successful results, rather than dealing with each problem individually, as has been done before in the literature. We identified significant problems as outlier, variable significance, noise, multicollinearity, and class imbalance. For these purposes, we created four different data sets according to the missing data situations. Thus, we were able to examine the changes in the interpretations of the models when outliers were removed. For each dataset, we created XGBoost models, which is a multicollinearity resistant method. While obtaining the performances of these models, we used the crossvalidation method. We used SMOTE and EF methods to deal with both noise and class imbalance problems separately and together in crossvalidation. As a result, the models we obtained were successful in more metrics when all identified problems were taken into account. We believe that this study will be useful to researchers who will do other disease detection modelling.

## Figures and Tables

**Algorithm 1:**
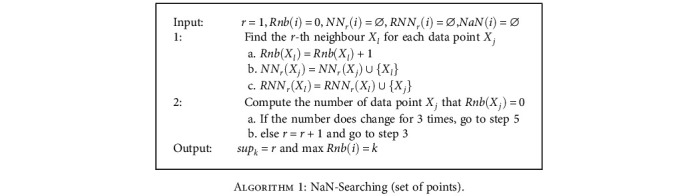
NaN-Searching (set of points).

**Algorithm 2:**
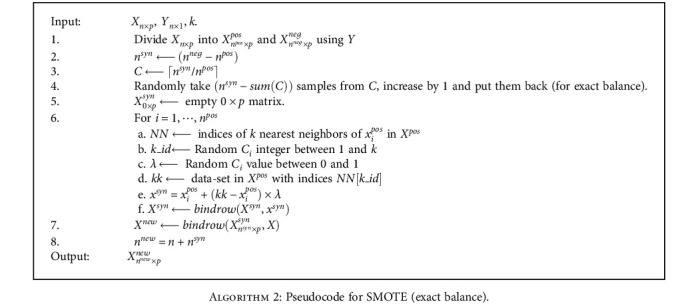
Pseudocode for SMOTE (exact balance).

**Table 1:** List of laboratory test abbreviations used in this study.

Laboratory test	Abbreviations
Hemoglobin	Hb
Hematocrit	Hct
Mean corpuscular volume	MCV
Mean corpuscular hemoglobin concentration	MCHC
Red blood cell count	RBC
Red blood cell distribution width	RDW
Iron	Fe
Unsaturated iron binding capacity	UIBC
Ferritin	FERR
Disease diagnosis	DD

**Table 2:** Confusion matrix.

Truth	Prediction	
	Positive	Negative
Positive	TP	FN
Negative	FP	TN

**Table 3:** Summary statistics of features in the datasets obtained and statistics for average person.

Feature	Outlier removal method									
	Original data		*Z*		RDOS		NOF		Average person	
	Min	Max	Min	Max	Min	Max	Min	Max	Min	Max
Age	17.00	89.00	17.00	88.00	18.00	82.00	17.00	89.00	—	—
Hb	6.20	17.30	6.20	15.80	6.20	15.80	6.20	17.30	11 gm/dL	18 gm/dL
Hct	18.30	48.80	21.00	46.30	21.00	46.30	18.30	48.80	35 perc	49 perc
MCV	34.90	111.60	52.50	98.90	52.50	96.00	34.90	99.70	80 fl	100 fl
MCHC	25.10	309.00	29.60	36.20	29.10	36.00	25.10	36.20	31 gr	37 gr
RBC	2.02	6.63	3.02	5.80	2.93	5.65	2.02	6.63	4.2 mcL	6.1 mcL
RDW	3.50	128.00	12.00	24.40	12.20	36.50	3.50	38.60	11.8 perc	16.1 perc
Fe	1.00	464.00	1.00	111.00	1.00	133.00	1.00	200.00	80 mcg/dL	180 mcg/dL
UIBC	4.78	717.00	135.00	634.00	145.00	591.00	126.00	591.00	111 mcg/dL	343 mcg/dL
FERR	1.00	1650.00	1.00	84.00	1.00	669.00	1.00	965.00	10 ng/mL	263 ng/mL

**Table 4:** Summary statistics for response variable.

Dataset	*n*	Outliers	True outliers	False outliers	True	False	Imbalance rate (true/false)
Original	516	0	0	0	359	157	2.287
*Z*	353	163	72	91	287	66	4.348
RDOS	269	247	162	85	197	72	2.736
NOF	488	28	23	5	336	152	2.211

**Table 5:** Best hyperparameters for XGBoost models.

	nrounds	Lambda	Alpha	Eta
Grid	(25, 50, 75, 100, 125, 150)	(0, 0.25, 0.5, 0.75, 1)	(0, 0.25, 0.5, 0.75, 1)	(0.01, 0.1, 0.25)
Original data				
No resampling	25	0	0.5	0.01
EF	50	0.75	0	0.01
SMOTE	50	0	0.75	0.01
EF + SMOTE	25	0.75	0.5	0.01
*Z*				
No resampling	50	0	0.25	0.01
EF	25	0.25	0.5	0.1
SMOTE	50	0	0.25	0.25
EF + SMOTE	75	0.5	1	0.01
RDOS				
No resampling	25	0.75	0.5	0.01
EF	50	0.25	0.25	0.1
SMOTE	25	0	0	0.1
EF + SMOTE	75	0	0	0.25
NOF				
No resampling	25	1	0.25	0.01
EF	25	0	0.25	0.01
SMOTE	25	0.5	0.5	0.01
EF + SMOTE	75	0.75	1	0.01

**Table 6:** Performance results for original data.

Metric	No resampling	EF	SMOTE	EF + SMOTE
ACC	0.9713 ± 0.0217	0.9727 ± 0.0207	0.9704 ± 0.0267	0.9735 ± 0.0214
MCC	0.9341 ± 0.0504	0.9378 ± 0.0470	0.9321 ± 0.0615	0.9400 ± 0.0479
AUC	0.9959 ± 0.0051	0.9952 ± 0.0058	0.9944 ± 0.0074	0.9933 ± 0.0087
Spec	0.9638 ± 0.0482	0.9732 ± 0.0366	0.9681 ± 0.0497	0.9764 ± 0.0373
Sens	0.9747 ± 0.0237	0.9725 ± 0.0252	0.9713 ± 0.0272	0.9721 ± 0.0271

**Table 7:** Performance results for *Z*.

Metric	No resampling	EF	SMOTE	EF + SMOTE
ACC	0.9878 ± 0.0202	0.9853 ± 0.0182	0.9898 ± 0.0160	0.9850 ± 0.0168
MCC	0.9616 ± 0.0645	0.9550 ± 0.0556	0.9676 ± 0.0527	0.9544 ± 0.0512
AUC	0.9981 ± 0.0046	0.9979 ± 0.0047	0.9986 ± 0.0032	0.9985 ± 0.0035
Spec	0.9733 ± 0.0684	0.9771 ± 0.0631	0.9779 ± 0.0647	0.9814 ± 0.0560
Sens	0.9913 ± 0.0180	0.9871 ± 0.0202	0.9923 ± 0.0145	0.9857 ± 0.0192

**Table 8:** Performance results for RDOS.

Metric	No resampling	EF	SMOTE	EF + SMOTE
ACC	0.9841 ± 0.0235	0.9773 ± 0.0308	0.9837 ± 0.0213	0.9786 ± 0.0291
MCC	0.9628 ± 0.0546	0.9482 ± 0.0660	0.9615 ± 0.0495	0.9508 ± 0.0642
AUC	0.9988 ± 0.0038	0.9958 ± 0.0093	0.9989 ± 0.0043	0.9952 ± 0.0101
Spec	0.9864 ± 0.0447	0.9863 ± 0.0415	0.9877 ± 0.0395	0.9889 ± 0.0378
Sens	0.9833 ± 0.0296	0.9741 ± 0.0410	0.9824 ± 0.0280	0.9747 ± 0.0382

**Table 9:** Performance results for NOF.

Metric	No resampling	EF	SMOTE	EF + SMOTE
ACC	0.9689 ± 0.0208	0.9723 ± 0.0228	0.9691 ± 0.0259	0.9729 ± 0.0251
MCC	0.9301 ± 0.0467	0.9383 ± 0.0497	0.9311 ± 0.0573	0.9395 ± 0.0558
AUC	0.9962 ± 0.0046	0.9955 ± 0.0090	0.9961 ± 0.0054	0.9947 ± 0.0078
Spec	0.9666 ± 0.0485	0.9709 ± 0.0456	0.9678 ± 0.0534	0.9757 ± 0.0425
Sens	0.9699 ± 0.0264	0.9729 ± 0.0304	0.9697 ± 0.0322	0.9717 ± 0.0300

## Data Availability

The data used to support the findings of this study are included within the supplementary information file(s).
