# Supplementary material for: A Novel Expert System for Diagnosis of Iron Deficiency Anemia
Source: Comput Math Methods Med. 2022 Oct 14;2022:7352096. doi: 10.1155/2022/7352096 (PMC9586777; doi:10.1155/2022/7352096)
Supplement: Supplementary Materials — Dataset and R codes used in the study are available as supplementary files online. [file 7352096.f1.zip › dataset.docx]

| Age | Hb | Hct | MCV | MCHC | RBC | RDW | Fe | UIBC | FERR | DD |
| --- | --- | --- | --- | --- | --- | --- | --- | --- | --- | --- |
| 38 | 11.7 | 34.7 | 84.7 | 33.79 | 4.09 | 14 | 28 | 376 | 10 | TRUE |
| 47 | 8.8 | 26.8 | 61.9 | 32.9 | 4.33 | 16.39 | 13 | 484 | 5 | TRUE |
| 36 | 9.19 | 26.3 | 78.4 | 35 | 3.35 | 14 | 54 | 255 | 99 | TRUE |
| 49 | 11.8 | 34.4 | 74.09 | 34.2 | 4.65 | 13.3 | 29 | 408 | 10 | TRUE |
| 46 | 9.9 | 30.1 | 71.3 | 32.9 | 4.23 | 26.2 | 259 | 144 | 10 | TRUE |
| 47 | 10.1 | 30.4 | 72.59 | 33.2 | 4.19 | 15.2 | 21 | 453 | 5 | TRUE |
| 46 | 9.19 | 27.7 | 62.8 | 33.29 | 4.41 | 18.6 | 10 | 443 | 10 | TRUE |
| 18 | 9.4 | 28.7 | 57.22 | 32.7 | 5.01 | 17.5 | 12 | 476 | 3 | TRUE |
| 33 | 8.8 | 27.2 | 56.6 | 32.2 | 4.8 | 18.89 | 8 | 441 | 2 | TRUE |
| 48 | 10.7 | 32 | 75.3 | 33.6 | 4.23 | 15 | 18 | 503 | 9 | TRUE |
| 30 | 10.9 | 31.6 | 76.8 | 34.29 | 4.11 | 14.8 | 18 | 337 | 16 | TRUE |
| 25 | 10.5 | 31.3 | 74.59 | 33.7 | 4.19 | 16.1 | 15 | 431 | 7 | TRUE |
| 59 | 10.3 | 30.07 | 77.8 | 33.6 | 3.95 | 23.5 | 14 | 97 | 1323 | TRUE |
| 36 | 7.4 | 23.1 | 57.5 | 31.8 | 4.01 | 18.5 | 8 | 479 | 2 | TRUE |
| 43 | 9.5 | 29.5 | 61.7 | 32.1 | 4.78 | 19.39 | 20 | 487 | 5 | TRUE |
| 64 | 9.5 | 28.6 | 75 | 33.4 | 3.81 | 16.39 | 25 | 428 | 45 | TRUE |
| 49 | 9.1 | 27.8 | 67.4 | 32.79 | 4.12 | 16.5 | 19 | 397 | 10 | TRUE |
| 60 | 9 | 27.1 | 73 | 33.29 | 3.71 | 17.1 | 26 | 486 | 8 | TRUE |
| 33 | 11.3 | 32.7 | 77.8 | 34.4 | 4.21 | 14.4 | 37 | 349 | 5 | TRUE |
| 51 | 7.3 | 22.7 | 70 | 32.29 | 3.24 | 19.7 | 5 | 485 | 3 | TRUE |
| 70 | 8.1 | 24.9 | 63.3 | 32.5 | 3.93 | 19.3 | 11 | 411 | 6 | TRUE |
| 34 | 11 | 33 | 73.2 | 33.29 | 4.51 | 16.6 | 34 | 375 | 9 | TRUE |
| 43 | 10.9 | 32.1 | 74.9 | 33.9 | 4.28 | 16.2 | 44 | 371 | 11 | TRUE |
| 22 | 11.1 | 33.1 | 67.8 | 33.6 | 4.88 | 17 | 20 | 358 | 12 | TRUE |
| 45 | 10.6 | 33.2 | 62.6 | 31.9 | 5.31 | 18.2 | 29 | 439 | 8 | TRUE |
| 45 | 10.6 | 31.5 | 70.59 | 33.6 | 4.46 | 17.2 | 23 | 403 | 5 | TRUE |
| 38 | 8.9 | 28 | 60.5 | 31.6 | 4.63 | 18.6 | 16 | 503 | 3 | TRUE |
| 31 | 9.5 | 28 | 90.7 | 34 | 3.09 | 15.2 | 32 | 497 | 3 | TRUE |
| 42 | 12.1 | 35.6 | 71.2 | 34.1 | 5 | 33.1 | 96 | 216 | 7 | TRUE |
| 36 | 12.1 | 35.9 | 74.8 | 33.79 | 4.8 | 15.6 | 44 | 392 | 9 | TRUE |
| 37 | 10.9 | 33.4 | 70.3 | 32.6 | 4.75 | 15.4 | 20 | 466 | 4 | TRUE |
| 40 | 13.4 | 39.1 | 86.01 | 34.4 | 4.54 | 14.1 | 57 | 347 | 8 | TRUE |
| 46 | 6.8 | 21.8 | 63 | 31.3 | 3.46 | 17.3 | 9 | 462 | 2 | TRUE |
| 84 | 11.5 | 34.1 | 78.5 | 33.6 | 4.34 | 19.3 | 21 | 273 | 34 | TRUE |
| 28 | 10.19 | 30.2 | 76.8 | 33.9 | 3.93 | 24.3 | 34 | 430 | 9 | TRUE |
| 35 | 9.4 | 28.4 | 68.4 | 33.29 | 4.13 | 20.3 | 15 | 418 | 3 | TRUE |
| 55 | 7.9 | 23.4 | 84.4 | 33.6 | 2.77 | 18.7 | 90 | 212 | 862 | TRUE |
| 36 | 9.19 | 28.9 | 61.7 | 31.7 | 4.69 | 20.1 | 12 | 531 | 3 | TRUE |
| 37 | 11 | 32.9 | 75.5 | 33.5 | 4.43 | 15.2 | 21 | 455 | 6 | TRUE |
| 78 | 9.9 | 29.3 | 73.4 | 33.79 | 3.99 | 15 | 17 | 291 | 2 | TRUE |
| 27 | 11.7 | 35.4 | 68.7 | 33.1 | 5.15 | 15.8 | 25 | 488 | 7 | TRUE |
| 68 | 12.1 | 35.2 | 88.5 | 34.4 | 3.98 | 14.3 | 55 | 236 | 65 | TRUE |
| 65 | 10.19 | 29.2 | 55 | 34.9 | 5.3 | 38.6 | 34 | 234 | 382 | TRUE |
| 47 | 10.1 | 30.2 | 65.5 | 33.4 | 4.59 | 17.89 | 24 | 424 | 6 | TRUE |
| 22 | 11.7 | 33.79 | 77.59 | 34.6 | 4.36 | 15.2 | 20 | 297 | 73 | TRUE |
| 34 | 12.7 | 37.2 | 77.3 | 34.1 | 4.82 | 14.4 | 44 | 292 | 17 | TRUE |
| 38 | 10.19 | 30.3 | 74.9 | 33.79 | 4.05 | 14.8 | 31 | 401 | 8 | TRUE |
| 76 | 11.9 | 35.7 | 85.6 | 33.4 | 4.17 | 16 | 36 | 401 | 9 | TRUE |
| 39 | 9.4 | 28.6 | 64.3 | 32.7 | 4.45 | 16 | 20 | 421 | 4 | TRUE |
| 45 | 13.4 | 38.6 | 83 | 34.6 | 4.65 | 14.6 | 36 | 307 | 21 | TRUE |
| 87 | 11.5 | 33.6 | 91.8 | 34.1 | 3.66 | 20.5 | 14 | 160 | 292 | TRUE |
| 51 | 14.1 | 40.6 | 86.8 | 34.7 | 4.68 | 15.4 | 62 | 288 | 16 | TRUE |
| 37 | 12.8 | 36.4 | 82.9 | 35.2 | 4.38 | 13.2 | 67 | 293 | 8 | TRUE |
| 43 | 9 | 27.5 | 68.3 | 32.7 | 4 | 17.7 | 18 | 373 | 6 | TRUE |
| 76 | 10.19 | 32.9 | 64.8 | 31.1 | 5 | 25.4 | 16 | 426 | 12 | TRUE |
| 39 | 9.5 | 29.4 | 66.59 | 32.29 | 4.41 | 23 | 18 | 349 | 84 | TRUE |
| 39 | 12.8 | 38.7 | 74.3 | 33.2 | 5.21 | 15.7 | 30 | 494 | 12 | TRUE |
| 30 | 10.9 | 32.7 | 82 | 33.4 | 3.98 | 15.2 | 17 | 379 | 4 | TRUE |
| 31 | 11.4 | 33.29 | 70.59 | 34.1 | 4.72 | 16.1 | 22 | 384 | 12 | TRUE |
| 50 | 7 | 22.4 | 58 | 31.3 | 3.86 | 18 | 12 | 504 | 4 | TRUE |
| 35 | 7.1 | 23.7 | 52.5 | 30 | 4.51 | 22.8 | 8 | 485 | 3 | TRUE |
| 38 | 12.7 | 38.9 | 79.59 | 32.5 | 4.88 | 17.3 | 61 | 336 | 13 | TRUE |
| 36 | 12 | 34.5 | 81.5 | 34.7 | 4.23 | 17.3 | 41 | 301 | 19 | TRUE |
| 27 | 10 | 29.4 | 65 | 33.9 | 4.51 | 16.2 | 13 | 404 | 34 | TRUE |
| 23 | 8.3 | 26.6 | 58 | 31.3 | 4.59 | 24.1 | 41 | 717 | 6 | TRUE |
| 37 | 7.8 | 24.9 | 71.59 | 31.4 | 3.48 | 22.9 | 200 | 186 | 8 | TRUE |
| 37 | 10.19 | 29 | 75.8 | 35.2 | 3.82 | 14.4 | 16 | 365 | 3 | TRUE |
| 73 | 8.9 | 28.2 | 65.09 | 31.4 | 4.33 | 18.6 | 10 | 485 | 4 | TRUE |
| 30 | 8.4 | 26.6 | 57.2 | 31.6 | 4.65 | 20.5 | 16 | 406 | 2 | TRUE |
| 39 | 11.3 | 34.9 | 58.5 | 32.2 | 5.98 | 18.3 | 44 | 394 | 12 | TRUE |
| 34 | 11.7 | 34.4 | 72.7 | 34.1 | 4.73 | 25.3 | 20 | 226 | 386 | TRUE |
| 34 | 8.1 | 23.7 | 81.5 | 34.4 | 2.9 | 15.6 | 311 | 68 | 16 | TRUE |
| 38 | 13.1 | 38.29 | 83.6 | 34.29 | 4.57 | 17.7 | 79 | 340 | 8 | TRUE |
| 63 | 11.1 | 33.5 | 78.3 | 33.29 | 4.26 | 24.6 | 67 | 372 | 486 | TRUE |
| 23 | 7.7 | 24.2 | 63 | 31.8 | 3.85 | 18.7 | 8 | 463 | 1 | TRUE |
| 41 | 7.5 | 24.1 | 56.6 | 31.2 | 4.26 | 21.2 | 9 | 444 | 2 | TRUE |
| 43 | 8.4 | 26.5 | 57.4 | 31.9 | 4.61 | 21.3 | 10 | 494 | 3 | TRUE |
| 66 | 10.7 | 31.1 | 76.09 | 34.5 | 4.08 | 18.89 | 45 | 347 | 4 | TRUE |
| 20 | 6.8 | 24.2 | 51.9 | 28.2 | 4.66 | 22.2 | 6 | 434 | 1 | TRUE |
| 44 | 10.1 | 30.8 | 72.3 | 32.9 | 4.26 | 17.8 | 24 | 324 | 33 | TRUE |
| 64 | 10.4 | 30.9 | 80.7 | 33.7 | 3.83 | 15.5 | 59 | 254 | 83 | TRUE |
| 73 | 9.8 | 29.7 | 76.4 | 33.1 | 3.89 | 16.8 | 28 | 403 | 8 | TRUE |
| 19 | 11.4 | 34.1 | 61.8 | 33.29 | 5.52 | 15.7 | 50 | 292 | 23 | TRUE |
| 34 | 13.4 | 38.6 | 79.09 | 34.6 | 4.88 | 32 | 72 | 183 | 174 | TRUE |
| 28 | 11.9 | 33.79 | 89.6 | 35.1 | 3.77 | 13.1 | 94 | 255 | 9 | TRUE |
| 62 | 13.7 | 40.9 | 81.4 | 33.6 | 5.01 | 25.6 | 57 | 258 | 581 | TRUE |
| 67 | 10.3 | 29.7 | 87.6 | 34.6 | 3.39 | 16.39 | 41 | 236 | 137 | TRUE |
| 21 | 8 | 25.8 | 56.3 | 30.8 | 4.59 | 19.1 | 9 | 483 | 1 | TRUE |
| 65 | 9.9 | 29.2 | 79.5 | 34.1 | 3.67 | 16 | 61 | 426 | 10 | TRUE |
| 51 | 10.5 | 30.7 | 78.9 | 34.2 | 3.89 | 15.7 | 34 | 384 | 14 | TRUE |
| 20 | 14.3 | 42 | 81.2 | 34.1 | 5.17 | 14.8 | 22 | 435 | 6 | TRUE |
| 51 | 11.3 | 33.9 | 70 | 33.4 | 4.84 | 16.89 | 21 | 477 | 4 | TRUE |
| 45 | 8.3 | 25.1 | 63.9 | 33.1 | 3.93 | 16.39 | 11 | 526 | 3 | TRUE |
| 38 | 10.19 | 31.3 | 63 | 32.5 | 4.97 | 19.5 | 10 | 468 | 5 | TRUE |
| 28 | 9 | 25.9 | 73.8 | 34.7 | 3.51 | 15.2 | 29 | 502 | 6 | TRUE |
| 51 | 9.69 | 30.06 | 64.3 | 31.3 | 4.76 | 17.5 | 46 | 266 | 108 | TRUE |
| 46 | 11.8 | 33.6 | 75.9 | 35.2 | 4.44 | 15.3 | 73 | 336 | 12 | TRUE |
| 35 | 10.9 | 32.4 | 77.3 | 33.5 | 4.19 | 13.5 | 28 | 582 | 7 | TRUE |
| 39 | 12.9 | 36.79 | 81.4 | 34.9 | 4.53 | 20.8 | 143 | 126 | 134 | TRUE |
| 76 | 8.8 | 24.4 | 111.6 | 36 | 2.19 | 12.1 | 36 | 204 | 338 | TRUE |
| 33 | 10.6 | 31.8 | 74.8 | 33.2 | 4.25 | 16.3 | 90 | 353 | 12 | TRUE |
| 47 | 11.5 | 35.29 | 82.9 | 32.5 | 4.26 | 16.1 | 19 | 245 | 7 | TRUE |
| 35 | 7.6 | 24 | 62.3 | 31.8 | 3.86 | 21 | 13 | 449 | 5 | TRUE |
| 54 | 11.6 | 34.2 | 75.9 | 33.79 | 4.5 | 13.8 | 21 | 253 | 82 | TRUE |
| 36 | 12.1 | 35.2 | 82.9 | 34.2 | 4.24 | 14.8 | 37 | 355 | 21 | TRUE |
| 37 | 9.1 | 27.9 | 59.4 | 32.5 | 4.69 | 19.3 | 9 | 380 | 3 | TRUE |
| 22 | 13.9 | 39.79 | 80.9 | 35.1 | 4.92 | 13.8 | 45 | 331 | 12 | TRUE |
| 66 | 10 | 28.7 | 80.5 | 34.7 | 3.57 | 14 | 39 | 265 | 30 | TRUE |
| 40 | 12.8 | 37.1 | 86 | 34.5 | 4.32 | 13.8 | 110 | 285 | 9 | TRUE |
| 25 | 10.1 | 31.6 | 66.9 | 32 | 4.72 | 16.9 | 26 | 416 | 3 | TRUE |
| 24 | 10.1 | 30.4 | 71.3 | 33.2 | 4.26 | 16.1 | 23 | 480 | 10 | TRUE |
| 66 | 10.1 | 29.5 | 91.4 | 34.29 | 3.23 | 15.4 | 64 | 228 | 79 | TRUE |
| 40 | 10.1 | 30.6 | 65.8 | 33 | 4.65 | 15.4 | 39 | 359 | 17 | TRUE |
| 51 | 8.8 | 27.2 | 66 | 32.2 | 4.12 | 21.5 | 19 | 442 | 5 | TRUE |
| 47 | 11.8 | 34.5 | 75.59 | 34.2 | 4.55 | 16.2 | 34 | 367 | 8 | TRUE |
| 49 | 12.7 | 37.7 | 85 | 33.6 | 4.44 | 19.3 | 25 | 332 | 13 | TRUE |
| 28 | 13.5 | 37.29 | 87.5 | 36.2 | 4.26 | 15.7 | 141 | 138 | 320 | TRUE |
| 51 | 9.4 | 29 | 69 | 32.29 | 4.2 | 17.3 | 10 | 513 | 4 | TRUE |
| 44 | 8.9 | 28.7 | 62.5 | 30.8 | 4.59 | 34.6 | 14 | 379 | 5 | TRUE |
| 46 | 11.5 | 33.4 | 81.9 | 34.5 | 4.07 | 13.9 | 29 | 439 | 7 | TRUE |
| 45 | 9.96 | 31.6 | 72.4 | 31.5 | 4.37 | 18.8 | 46 | 363 | 4 | TRUE |
| 51 | 12.9 | 36.9 | 81.7 | 35 | 4.51 | 19.6 | 89 | 192 | 199 | TRUE |
| 48 | 10.5 | 29.4 | 80.2 | 35.7 | 3.66 | 13.6 | 29 | 333 | 9 | TRUE |
| 39 | 8.9 | 27.3 | 60.6 | 32.6 | 4.51 | 19.89 | 15 | 520 | 3 | TRUE |
| 39 | 12.8 | 37.2 | 83.7 | 34.4 | 4.45 | 16 | 46 | 267 | 139 | TRUE |
| 67 | 13.8 | 40.79 | 78.2 | 33.9 | 5.22 | 15.4 | 66 | 357 | 7 | TRUE |
| 31 | 8.6 | 26.5 | 65.8 | 32.5 | 4.01 | 17.8 | 9 | 453 | 1 | TRUE |
| 32 | 11.7 | 35.5 | 62.9 | 33 | 5.65 | 18.7 | 13 | 400 | 16 | TRUE |
| 38 | 10.3 | 31.7 | 76.4 | 32.3 | 4.16 | 16.9 | 47 | 407 | 10 | TRUE |
| 48 | 10.7 | 31.9 | 72.9 | 33.5 | 4.37 | 33 | 35 | 313 | 51 | TRUE |
| 41 | 6.4 | 21.1 | 60.4 | 29.1 | 3.66 | 24.4 | 7 | 466 | 1 | TRUE |
| 44 | 10.9 | 35.3 | 75.6 | 309 | 4.67 | 46.9 | 20 | 343 | 8 | TRUE |
| 42 | 10.7 | 32.4 | 73.7 | 33.2 | 4.38 | 16.89 | 13 | 421 | 8 | TRUE |
| 75 | 7.5 | 24.1 | 79.59 | 31.2 | 3.02 | 20.8 | 8 | 337 | 6 | TRUE |
| 36 | 8.69 | 27.1 | 65.2 | 32.1 | 4.16 | 19.39 | 15 | 523 | 3 | TRUE |
| 25 | 10.7 | 32.79 | 68.4 | 32.6 | 4.79 | 16.7 | 19 | 426 | 3 | TRUE |
| 26 | 10.9 | 33.1 | 75.2 | 32.9 | 4.4 | 15.9 | 121 | 145 | 669 | TRUE |
| 45 | 8.19 | 26.3 | 59.4 | 31.2 | 4.43 | 20.7 | 6 | 466 | 3 | TRUE |
| 42 | 7.4 | 23.6 | 56 | 31.3 | 4.21 | 21.1 | 12 | 580 | 4 | TRUE |
| 24 | 7 | 23.2 | 54.5 | 30.2 | 4.26 | 19.8 | 13 | 465 | 2 | TRUE |
| 41 | 11.7 | 34.29 | 80.09 | 34.1 | 4.28 | 15.2 | 35 | 380 | 4 | TRUE |
| 67 | 10.4 | 31.8 | 77.8 | 32.9 | 4.08 | 18.8 | 464 | 514 | 15 | TRUE |
| 54 | 11.4 | 32.7 | 86.6 | 34.9 | 3.77 | 13.1 | 51 | 356 | 35 | TRUE |
| 36 | 10.1 | 30.9 | 64.9 | 32.7 | 4.76 | 17 | 17 | 333 | 7 | TRUE |
| 70 | 11.7 | 33.29 | 80.4 | 35.2 | 4.13 | 14.1 | 48 | 200 | 547 | TRUE |
| 36 | 7.9 | 24 | 63.6 | 32.79 | 3.77 | 16.8 | 15 | 432 | 3 | TRUE |
| 23 | 13.6 | 39.9 | 85.8 | 34.2 | 4.65 | 17 | 42 | 204 | 472 | TRUE |
| 20 | 13.4 | 39 | 78.8 | 34.5 | 4.95 | 24.7 | 138 | 270 | 242 | TRUE |
| 36 | 8.3 | 26.8 | 62.6 | 31.2 | 4.26 | 17.2 | 12 | 510 | 7 | TRUE |
| 36 | 9.4 | 29 | 68.3 | 32.5 | 4.24 | 16.8 | 26 | 344 | 4 | TRUE |
| 43 | 13.7 | 39.1 | 84.8 | 35.1 | 4.61 | 17.1 | 43 | 223 | 116 | TRUE |
| 63 | 9.8 | 28.9 | 77.8 | 33.9 | 3.71 | 17.39 | 77 | 223 | 525 | TRUE |
| 46 | 8.9 | 27.6 | 64.5 | 32.3 | 4.28 | 17.7 | 15 | 467 | 3 | TRUE |
| 45 | 12.9 | 37.9 | 77.59 | 34 | 4.88 | 18.5 | 37 | 404 | 14 | TRUE |
| 61 | 8.69 | 27.1 | 67.59 | 32.1 | 4.01 | 21.5 | 27 | 510 | 6 | TRUE |
| 44 | 7.8 | 24.4 | 58.8 | 31.9 | 4.13 | 18.7 | 20 | 407 | 3 | TRUE |
| 44 | 8.9 | 27.2 | 65.09 | 32.7 | 4.19 | 20.3 | 17 | 488 | 145 | TRUE |
| 24 | 10.4 | 31.2 | 73.2 | 33.5 | 4.26 | 15.6 | 22 | 432 | 4 | TRUE |
| 31 | 10.5 | 33.1 | 73.8 | 31.8 | 4.48 | 19.39 | 53 | 313 | 501 | TRUE |
| 37 | 11.2 | 32.9 | 84.2 | 34.2 | 3.9 | 13.9 | 50 | 307 | 8 | TRUE |
| 36 | 7.8 | 25.8 | 58.4 | 30.3 | 4.43 | 17.89 | 12 | 516 | 1 | TRUE |
| 39 | 6.5 | 18.3 | 90 | 35.5 | 2.02 | 12.5 | 58 | 276 | 371 | TRUE |
| 78 | 8.9 | 26.4 | 90.6 | 33.5 | 2.92 | 16.89 | 32 | 359 | 42 | TRUE |
| 76 | 9.9 | 29.9 | 81.7 | 33.1 | 3.66 | 17.7 | 25 | 218 | 78 | TRUE |
| 49 | 11.6 | 33.9 | 75 | 34.1 | 4.53 | 12.8 | 24 | 436 | 9 | TRUE |
| 30 | 10.7 | 32.7 | 73.8 | 32.79 | 4.43 | 17.89 | 23 | 447 | 3 | TRUE |
| 41 | 11.1 | 33.6 | 67.9 | 32.9 | 4.95 | 16.3 | 9 | 466 | 4 | TRUE |
| 50 | 9.5 | 28.9 | 68.09 | 32.79 | 4.25 | 15.8 | 15 | 435 | 9 | TRUE |
| 39 | 10.7 | 32.9 | 60.9 | 32.5 | 5.41 | 16.5 | 50 | 280 | 8 | TRUE |
| 39 | 11.9 | 34.9 | 81.2 | 34.2 | 4.3 | 18.6 | 33 | 373 | 25 | TRUE |
| 57 | 12.7 | 37.8 | 79 | 33.7 | 4.78 | 15.9 | 36 | 384 | 7 | TRUE |
| 32 | 10.8 | 32.3 | 79.3 | 33.5 | 4.07 | 14.3 | 32 | 313 | 34 | TRUE |
| 30 | 10.2 | 31 | 65.4 | 32.9 | 4.75 | 35.5 | 13 | 421 | 16 | TRUE |
| 44 | 9.9 | 31.3 | 67.3 | 31.5 | 4.65 | 15.5 | 15 | 462 | 3 | TRUE |
| 28 | 9.9 | 31.7 | 62.6 | 31.4 | 5.06 | 19.2 | 14 | 516 | 3 | TRUE |
| 34 | 9.1 | 28.6 | 66.5 | 31.8 | 4.31 | 20.4 | 11 | 444 | 4 | TRUE |
| 41 | 12 | 36.5 | 74.1 | 32.9 | 4.92 | 16.9 | 29 | 496 | 15 | TRUE |
| 26 | 14.4 | 41.9 | 69 | 34.29 | 6.06 | 14.9 | 44 | 414 | 18 | TRUE |
| 25 | 8.6 | 26.6 | 65 | 32.2 | 4.09 | 20.9 | 9 | 460 | 7 | TRUE |
| 43 | 7.6 | 24.9 | 57.8 | 30.3 | 4.31 | 19.4 | 4 | 542 | 1 | TRUE |
| 58 | 13.7 | 43 | 85.7 | 31.8 | 5.02 | 21.6 | 34 | 308 | 24 | TRUE |
| 20 | 8.8 | 29.6 | 54.9 | 29.6 | 5.39 | 20.5 | 12 | 135 | 6 | TRUE |
| 37 | 6.2 | 21 | 56.9 | 29.7 | 3.69 | 19.6 | 1 | 457 | 4 | TRUE |
| 51 | 8.9 | 25.3 | 80.1 | 35.1 | 3.16 | 18.1 | 18 | 136 | 1650 | TRUE |
| 42 | 10.3 | 32.1 | 64.9 | 32.1 | 4.94 | 17.4 | 30 | 435 | 22 | TRUE |
| 48 | 8.8 | 27.4 | 65.7 | 32 | 4.17 | 16 | 6 | 457 | 4 | TRUE |
| 46 | 10.2 | 31.2 | 70.9 | 32.6 | 4.41 | 17.2 | 19 | 415 | 4 | TRUE |
| 40 | 11.8 | 34.8 | 76.3 | 33.8 | 4.56 | 17 | 30 | 374 | 14 | TRUE |
| 47 | 15.8 | 48.2 | 72.7 | 32.8 | 6.63 | 14.3 | 21 | 328 | 30 | TRUE |
| 44 | 8.6 | 26.2 | 70.1 | 32.9 | 3.74 | 17.8 | 13 | 445 | 2 | TRUE |
| 60 | 8.6 | 25.4 | 82.1 | 33.9 | 3.09 | 16.4 | 43 | 145 | 341 | TRUE |
| 44 | 14.2 | 42.6 | 89.2 | 33.3 | 4.77 | 14.6 | 31 | 345 | 32 | TRUE |
| 68 | 9.3 | 29.4 | 70 | 31.7 | 4.2 | 17 | 34 | 214 | 723 | TRUE |
| 45 | 10.9 | 32.8 | 76.1 | 33.1 | 4.31 | 16 | 30 | 395 | 4 | TRUE |
| 52 | 12.8 | 40 | 75.6 | 32.1 | 5.29 | 36.5 | 52 | 355 | 25 | TRUE |
| 39 | 9.3 | 30.1 | 57.8 | 31 | 5.21 | 22.8 | 10 | 396 | 2 | TRUE |
| 31 | 9.3 | 29.3 | 65.6 | 31.8 | 4.47 | 18.5 | 11 | 402 | 3 | TRUE |
| 43 | 7.2 | 24 | 60.2 | 30.1 | 3.99 | 19 | 11 | 327 | 7 | TRUE |
| 46 | 13.9 | 41.4 | 84.7 | 33.7 | 4.89 | 12.8 | 29 | 343 | 7 | TRUE |
| 54 | 10.2 | 30 | 79.8 | 34 | 3.76 | 15.5 | 52 | 413 | 6 | TRUE |
| 40 | 8.1 | 25.9 | 57.1 | 31.7 | 4.53 | 22.4 | 11 | 474 | 3 | TRUE |
| 53 | 12 | 37.7 | 72.1 | 31.9 | 5.22 | 30.9 | 52 | 356 | 11 | TRUE |
| 45 | 10.5 | 32.2 | 76.2 | 32.7 | 4.23 | 17.7 | 10 | 430 | 6 | TRUE |
| 45 | 15.3 | 45.9 | 81.1 | 33.4 | 5.66 | 14.7 | 18 | 299 | 13 | TRUE |
| 60 | 15.5 | 46.3 | 82.8 | 33.6 | 5.59 | 15.6 | 30 | 344 | 22 | TRUE |
| 42 | 11.6 | 34 | 74.7 | 34.1 | 4.56 | 14.4 | 20 | 406 | 6 | TRUE |
| 59 | 10.8 | 32.4 | 82 | 33.3 | 3.96 | 14.1 | 29 | 368 | 46 | TRUE |
| 49 | 11.2 | 35.9 | 62.7 | 31.1 | 5.72 | 29 | 21 | 439 | 5 | TRUE |
| 40 | 11.4 | 35.2 | 68.9 | 32.2 | 5.11 | 24.7 | 13 | 406 | 13 | TRUE |
| 48 | 7.8 | 26 | 61 | 29.9 | 4.27 | 20.1 | 9 | 387 | 19 | TRUE |
| 38 | 14.5 | 43 | 81.7 | 33.7 | 5.26 | 13.9 | 51 | 391 | 7 | TRUE |
| 49 | 9.4 | 29.2 | 64.1 | 32.2 | 4.56 | 19.5 | 20 | 419 | 1 | TRUE |
| 37 | 12.9 | 37.4 | 71.9 | 34.6 | 5.21 | 14.4 | 32 | 337 | 8 | TRUE |
| 42 | 9.6 | 29.6 | 65.7 | 32.3 | 4.51 | 18.5 | 23 | 434 | 4 | TRUE |
| 40 | 11.7 | 35.5 | 82 | 33 | 4.33 | 15 | 49 | 405 | 5 | TRUE |
| 26 | 10.8 | 31.9 | 77.4 | 33.8 | 4.12 | 15.1 | 26 | 405 | 6 | TRUE |
| 39 | 9.2 | 28.4 | 71.3 | 32.5 | 3.98 | 15.8 | 18 | 418 | 3 | TRUE |
| 25 | 12.2 | 36 | 83.6 | 33.9 | 4.31 | 13.7 | 33 | 263 | 18 | TRUE |
| 18 | 11 | 33.2 | 55.1 | 33.1 | 6.02 | 19.9 | 28 | 370 | 23 | TRUE |
| 34 | 9.7 | 29.9 | 73.5 | 32.6 | 4.07 | 18 | 13 | 476 | 3 | TRUE |
| 18 | 11.7 | 34.1 | 80.3 | 34.4 | 4.25 | 14.5 | 15 | 302 | 24 | TRUE |
| 27 | 12.6 | 37.2 | 79.9 | 33.8 | 4.65 | 14 | 34 | 390 | 6 | TRUE |
| 51 | 10.3 | 31.8 | 71.2 | 32.2 | 4.47 | 19 | 15 | 479 | 6 | TRUE |
| 49 | 8.5 | 26.9 | 72.5 | 31.5 | 3.71 | 19.5 | 5 | 492 | 4 | TRUE |
| 44 | 11 | 33.8 | 77 | 32.5 | 4.39 | 17.4 | 12 | 366 | 6 | TRUE |
| 42 | 10.7 | 31.8 | 74.2 | 33.8 | 4.28 | 16.5 | 53 | 544 | 4 | TRUE |
| 41 | 9.6 | 29.5 | 66.5 | 32.6 | 4.43 | 20.5 | 22 | 434 | 8 | TRUE |
| 41 | 7.5 | 23.3 | 58.3 | 32.2 | 3.99 | 18.5 | 11 | 492 | 3 | TRUE |
| 68 | 10.4 | 31.8 | 67.7 | 32.6 | 4.71 | 18.89 | 16 | 441 | 6 | TRUE |
| 46 | 10.4 | 31.1 | 71.4 | 33.4 | 4.35 | 17.4 | 143 | 265 | 7 | TRUE |
| 38 | 9 | 27.7 | 66.3 | 32.4 | 4.18 | 17.1 | 19 | 365 | 2 | TRUE |
| 62 | 10.2 | 29.7 | 69.5 | 34.2 | 4.28 | 16.9 | 25 | 429 | 10 | TRUE |
| 49 | 11.1 | 33.2 | 71.7 | 33.6 | 4.63 | 29.6 | 32 | 384 | 6 | TRUE |
| 48 | 14.8 | 42.5 | 77.6 | 34.8 | 5.48 | 15.1 | 31 | 468 | 6 | TRUE |
| 41 | 11.6 | 34.8 | 71.8 | 33.4 | 4.85 | 20 | 29 | 438 | 4 | TRUE |
| 85 | 8.8 | 27.1 | 60 | 32.4 | 4.51 | 21.7 | 16 | 474 | 6 | TRUE |
| 71 | 7.9 | 25.3 | 69.2 | 31.4 | 3.66 | 17.5 | 5 | 449 | 4 | TRUE |
| 53 | 9.81 | 27.7 | 81.5 | 35.4 | 3.4 | 13.8 | 39 | 286 | 7 | TRUE |
| 40 | 10.8 | 34.5 | 77.8 | 31.3 | 4.43 | 27.8 | 23 | 273 | 137 | TRUE |
| 47 | 11.1 | 33.7 | 67.1 | 32.9 | 5.01 | 28.9 | 42 | 412 | 20 | TRUE |
| 66 | 10.8 | 32.1 | 80 | 33.8 | 4.01 | 15.4 | 16 | 398 | 3 | TRUE |
| 21 | 11.6 | 32.4 | 81.1 | 35.6 | 4 | 14.9 | 45 | 385 | 6 | TRUE |
| 23 | 9 | 27.8 | 59.8 | 32.5 | 4.65 | 19.8 | 14 | 455 | 3 | TRUE |
| 27 | 11.2 | 32.7 | 74.5 | 34.4 | 4.39 | 31 | 17 | 320 | 43 | TRUE |
| 38 | 7.4 | 23 | 62.7 | 32.3 | 3.67 | 23.3 | 9 | 417 | 3 | TRUE |
| 46 | 13.3 | 37.6 | 82.7 | 35.3 | 4.55 | 12.8 | 38 | 344 | 9 | TRUE |
| 45 | 9.6 | 28.1 | 70.3 | 34.2 | 3.99 | 18.3 | 17 | 448 | 113 | TRUE |
| 40 | 11.9 | 34.4 | 88.9 | 34.4 | 3.87 | 14 | 78 | 350 | 4 | TRUE |
| 60 | 9.6 | 29.7 | 62.2 | 32.3 | 4.77 | 24.1 | 41 | 371 | 20 | TRUE |
| 35 | 11.1 | 33.7 | 73.6 | 32.9 | 4.58 | 16.3 | 25 | 461 | 5 | TRUE |
| 41 | 7.8 | 25.1 | 54.1 | 30.9 | 4.64 | 23.8 | 12 | 466 | 2 | TRUE |
| 49 | 8.7 | 28.3 | 59.3 | 30.8 | 4.78 | 19.3 | 14 | 525 | 4 | TRUE |
| 28 | 11.5 | 33.6 | 80.3 | 34.3 | 4.18 | 23.7 | 48 | 393 | 22 | TRUE |
| 71 | 10.1 | 31.1 | 64.4 | 32.4 | 4.83 | 31.8 | 22 | 508 | 4 | TRUE |
| 73 | 8.3 | 25.1 | 71.9 | 33.1 | 3.49 | 16.8 | 10 | 453 | 8 | TRUE |
| 70 | 10.6 | 33 | 74.7 | 32.2 | 4.42 | 27.3 | 66 | 277 | 965 | TRUE |
| 62 | 12.6 | 39.1 | 66.3 | 32.3 | 5.89 | 16.7 | 39 | 388 | 16 | TRUE |
| 26 | 8 | 25.6 | 55.7 | 31.2 | 4.59 | 18.5 | 7 | 670 | 14 | TRUE |
| 26 | 9.2 | 28.4 | 72.1 | 32.3 | 3.94 | 20.8 | 13 | 528 | 2 | TRUE |
| 26 | 10.7 | 30.7 | 68.5 | 35 | 4.49 | 20.4 | 33 | 368 | 15 | TRUE |
| 33 | 10.4 | 31.3 | 69.5 | 33.4 | 4.5 | 30.2 | 23 | 464 | 16 | TRUE |
| 29 | 7.8 | 24.9 | 62.9 | 31.3 | 3.95 | 17.8 | 10 | 472 | 1 | TRUE |
| 35 | 9 | 27.7 | 66.3 | 32.4 | 4.18 | 17.1 | 19 | 365 | 3 | TRUE |
| 32 | 11.7 | 36.2 | 62.4 | 32.3 | 5.8 | 17.3 | 43 | 435 | 5 | TRUE |
| 36 | 12.6 | 35.6 | 84.1 | 35.4 | 4.23 | 14 | 48 | 377 | 9 | TRUE |
| 35 | 13.5 | 38.3 | 77.6 | 35.3 | 4.94 | 14.5 | 31 | 393 | 6 | TRUE |
| 37 | 10.5 | 31.1 | 70.4 | 33.9 | 4.42 | 15.1 | 25 | 521 | 3 | TRUE |
| 35 | 11.4 | 34.5 | 68.6 | 32.9 | 5.02 | 20.7 | 42 | 591 | 7 | TRUE |
| 36 | 12.3 | 35.3 | 82.3 | 34.8 | 4.29 | 15.2 | 45 | 634 | 10 | TRUE |
| 40 | 8.3 | 26.3 | 62.7 | 31.6 | 4.2 | 18.9 | 11 | 520 | 5 | TRUE |
| 37 | 8.4 | 26.4 | 58.3 | 31.7 | 4.52 | 19.1 | 12 | 484 | 1 | TRUE |
| 39 | 11.1 | 33.6 | 72.1 | 33.1 | 4.66 | 15.4 | 16 | 539 | 4 | TRUE |
| 49 | 9.2 | 30.1 | 60.8 | 30.5 | 4.95 | 21.4 | 19 | 479 | 7 | TRUE |
| 40 | 11.7 | 34.1 | 74.4 | 34.3 | 4.59 | 14 | 20 | 449 | 4 | TRUE |
| 52 | 10.7 | 33 | 69.4 | 32.4 | 4.79 | 16.6 | 17 | 400 | 8 | TRUE |
| 52 | 8 | 26.2 | 55 | 30.7 | 4.76 | 21.3 | 9 | 488 | 3 | TRUE |
| 43 | 7.8 | 24.8 | 60.3 | 31.3 | 4.11 | 20.9 | 10 | 425 | 2 | TRUE |
| 56 | 12.6 | 36.4 | 76.7 | 34.6 | 4.74 | 16.1 | 36 | 341 | 10 | TRUE |
| 52 | 11.5 | 33.1 | 82.6 | 34.7 | 4.01 | 18.8 | 26 | 369 | 9 | TRUE |
| 57 | 10.3 | 29.3 | 94.5 | 35.3 | 3.1 | 25.8 | 40 | 198 | 144 | TRUE |
| 52 | 9.8 | 30.6 | 70.1 | 32.2 | 4.36 | 18.9 | 27 | 463 | 3 | TRUE |
| 76 | 9.9 | 30.1 | 82.3 | 32.8 | 3.66 | 16.7 | 29 | 262 | 4 | TRUE |
| 53 | 10.3 | 30.6 | 79.4 | 33.7 | 3.85 | 13.3 | 70 | 338 | 20 | TRUE |
| 57 | 10.2 | 30.9 | 74.2 | 33.1 | 4.17 | 18.9 | 17 | 414 | 11 | TRUE |
| 66 | 13.9 | 40.9 | 81.9 | 34.1 | 4.99 | 14.7 | 29 | 356 | 17 | TRUE |
| 58 | 11.7 | 34.3 | 81 | 34.2 | 4.23 | 19.3 | 20 | 313 | 121 | TRUE |
| 68 | 9.4 | 28.6 | 63.2 | 33.1 | 4.52 | 16.6 | 21 | 396 | 13 | TRUE |
| 63 | 10.1 | 29.6 | 79.3 | 34.2 | 3.73 | 18.9 | 33 | 405 | 18 | TRUE |
| 28 | 11.7 | 35.1 | 61.8 | 33.3 | 5.68 | 17.1 | 51 | 474 | 7 | TRUE |
| 49 | 8 | 26.8 | 59.8 | 29.8 | 4.49 | 22.7 | 9 | 427 | 1 | TRUE |
| 39 | 7.2 | 23.5 | 57 | 30.5 | 4.12 | 19.4 | 12 | 4.78 | 1 | TRUE |
| 35 | 7.3 | 23.1 | 60.7 | 31.7 | 3.81 | 21.8 | 10 | 448 | 2 | TRUE |
| 26 | 10.7 | 31.6 | 74.4 | 33.79 | 4.25 | 15.4 | 26 | 385 | 5 | TRUE |
| 39 | 11.6 | 33.6 | 79.2 | 34.5 | 4.24 | 13 | 28 | 435 | 6 | TRUE |
| 64 | 9.9 | 29 | 78 | 34.1 | 3.71 | 15.9 | 19 | 155 | 282 | TRUE |
| 55 | 10.6 | 31.8 | 72.59 | 33.5 | 4.38 | 22.7 | 21 | 386 | 24 | TRUE |
| 71 | 10.19 | 29.6 | 82.5 | 34.4 | 3.59 | 15.3 | 34 | 299 | 28 | TRUE |
| 56 | 10.4 | 29.8 | 81.4 | 35 | 3.66 | 14.5 | 49 | 378 | 25 | TRUE |
| 49 | 9.3 | 28.7 | 64.8 | 32.4 | 4.43 | 18.39 | 11 | 500 | 7 | TRUE |
| 44 | 7.9 | 25.6 | 64.5 | 30.9 | 3.97 | 20.1 | 13 | 494 | 7 | TRUE |
| 44 | 8.69 | 28.3 | 60.9 | 30.7 | 4.63 | 20.6 | 10 | 494 | 4 | TRUE |
| 40 | 10.7 | 32.29 | 66.7 | 33.1 | 4.84 | 16.7 | 12 | 415 | 7 | TRUE |
| 38 | 8.9 | 26.9 | 71.59 | 33.29 | 3.76 | 16 | 20 | 392 | 5 | TRUE |
| 38 | 10.3 | 31.4 | 66.3 | 33 | 4.73 | 18.3 | 10 | 429 | 6 | TRUE |
| 22 | 8.9 | 28.3 | 60.9 | 31.6 | 4.63 | 17.8 | 17 | 466 | 4 | TRUE |
| 18 | 9.1 | 28.1 | 66.8 | 32.6 | 4.2 | 16 | 11 | 369 | 8 | TRUE |
| 75 | 9.4 | 27.3 | 80.7 | 34.1 | 3.39 | 16.3 | 21 | 183 | 178 | TRUE |
| 73 | 10.4 | 30.9 | 80.59 | 33.6 | 3.83 | 15.8 | 14 | 372 | 13 | TRUE |
| 66 | 8.1 | 25.6 | 58.5 | 31.7 | 4.38 | 26.6 | 24 | 405 | 12 | TRUE |
| 57 | 8.8 | 25.1 | 94.8 | 34.9 | 2.65 | 15.1 | 24 | 163 | 1154 | TRUE |
| 52 | 9.8 | 29.4 | 71.9 | 33.4 | 4.08 | 17 | 17 | 444 | 5 | TRUE |
| 40 | 12.1 | 34.2 | 80.59 | 35.5 | 4.24 | 14.9 | 22 | 407 | 10 | TRUE |
| 39 | 10.19 | 31.5 | 66.3 | 32.5 | 4.74 | 21.8 | 21 | 365 | 7 | TRUE |
| 46 | 6.7 | 21.8 | 58.9 | 30.7 | 3.7 | 24.4 | 10 | 427 | 4 | TRUE |
| 26 | 10.5 | 32.4 | 69.3 | 32.4 | 4.68 | 16.89 | 8 | 496 | 5 | TRUE |
| 20 | 11.6 | 34.7 | 80.5 | 33.5 | 4.3 | 15.6 | 22 | 391 | 5 | TRUE |
| 18 | 11.8 | 36.4 | 67.3 | 32.4 | 5.41 | 21.8 | 29 | 333 | 8 | TRUE |
| 17 | 8.2 | 26.5 | 60.8 | 30.7 | 4.37 | 17.8 | 11 | 444 | 3 | TRUE |
| 25 | 11.6 | 34.6 | 75.2 | 33.5 | 4.6 | 15.5 | 47 | 340 | 6 | TRUE |
| 31 | 11.3 | 33.5 | 71.8 | 33.7 | 4.66 | 21.7 | 21 | 400 | 8 | TRUE |
| 32 | 9.6 | 29.3 | 68.9 | 32.7 | 4.25 | 17.2 | 18 | 433 | 9 | TRUE |
| 38 | 11.3 | 33.2 | 78.4 | 34 | 4.23 | 22.6 | 40 | 342 | 7 | TRUE |
| 39 | 8.4 | 27 | 60.1 | 31.2 | 4.5 | 19.4 | 9 | 416 | 2 | TRUE |
| 41 | 9.3 | 29 | 66.8 | 32.1 | 4.33 | 16 | 21 | 404 | 9 | TRUE |
| 44 | 10.4 | 30.9 | 67.6 | 33.8 | 4.57 | 16.6 | 8 | 447 | 6 | TRUE |
| 47 | 8.6 | 25.3 | 86.2 | 33.9 | 2.93 | 16.9 | 30 | 346 | 10 | TRUE |
| 28 | 8.9 | 27.9 | 63 | 31.9 | 4.44 | 18 | 49 | 430 | 25 | TRUE |
| 31 | 12.7 | 37.2 | 88 | 34 | 4.22 | 19 | 50 | 399 | 6 | TRUE |
| 40 | 11.8 | 35.6 | 82.1 | 33.1 | 3.34 | 15.7 | 43 | 476 | 10 | TRUE |
| 40 | 10.5 | 31.1 | 69 | 33.8 | 4.52 | 15.4 | 23 | 369 | 17 | TRUE |
| 44 | 8.5 | 26.9 | 64.8 | 31.5 | 4.15 | 19.9 | 17 | 506 | 2 | TRUE |
| 45 | 12.4 | 37 | 74.2 | 33.6 | 4.99 | 15.5 | 24 | 370 | 33 | TRUE |
| 25 | 9.7 | 29.7 | 75.5 | 32.6 | 3.94 | 15.7 | 37 | 557 | 8 | TRUE |
| 30 | 10.6 | 31.7 | 83.4 | 33.7 | 3.79 | 16.1 | 59 | 426 | 5 | TRUE |
| 30 | 11.6 | 35.2 | 73.8 | 32.8 | 4.78 | 16.5 | 16 | 397 | 6 | TRUE |
| 42 | 9.7 | 29.4 | 66.3 | 33.1 | 4.43 | 17.3 | 6 | 452 | 3 | TRUE |
| 43 | 9.9 | 30.7 | 59.9 | 32.1 | 5.12 | 21.4 | 142 | 251 | 155 | TRUE |
| 45 | 7.6 | 24.7 | 71.6 | 31.2 | 3.42 | 18.7 | 16 | 458 | 4 | TRUE |
| 48 | 10 | 30.6 | 73.2 | 32.9 | 4.19 | 27.6 | 34 | 297 | 12 | TRUE |
| 48 | 9.1 | 28.5 | 66.6 | 32 | 4.28 | 17.8 | 14 | 449 | 4 | TRUE |
| 52 | 13.1 | 39.5 | 85.1 | 33.8 | 4.65 | 16.3 | 28 | 372 | 10 | TRUE |
| 55 | 8.3 | 26.6 | 61.6 | 31 | 4.35 | 27.5 | 15 | 514 | 5 | TRUE |
| 66 | 12 | 36.2 | 70.7 | 33.1 | 4.54 | 14.4 | 43 | 416 | 7 | TRUE |
| 20 | 10.4 | 31.9 | 65.2 | 32.6 | 4.89 | 17 | 11 | 427 | 3 | TRUE |
| 21 | 13.1 | 39.6 | 71.4 | 33.1 | 5.5 | 14.5 | 49 | 406 | 6 | TRUE |
| 22 | 10.4 | 30.6 | 69.5 | 34.2 | 4.4 | 17.4 | 60 | 425 | 4 | TRUE |
| 22 | 11.8 | 36.6 | 76.5 | 32.2 | 4.78 | 13.9 | 12 | 405 | 4 | TRUE |
| 34 | 10.3 | 32.3 | 68.7 | 31.8 | 4.7 | 20 | 18 | 390 | 8 | TRUE |
| 36 | 11.1 | 33.5 | 67.2 | 33 | 4.99 | 18.2 | 25 | 420 | 6 | TRUE |
| 38 | 8.2 | 26.9 | 57.6 | 31.3 | 4.55 | 18.1 | 3 | 457 | 3 | TRUE |
| 39 | 9.4 | 28.5 | 61.8 | 32.9 | 4.62 | 19.6 | 9 | 368 | 7 | TRUE |
| 39 | 10.6 | 32.2 | 74.1 | 33 | 4.34 | 15.4 | 13 | 452 | 4 | TRUE |
| 40 | 10.1 | 30.3 | 75.9 | 33.3 | 3.99 | 16.5 | 19 | 432 | 8 | TRUE |
| 45 | 11.6 | 33.2 | 73.4 | 35.1 | 4.53 | 16.4 | 24 | 413 | 6 | TRUE |
| 45 | 7.9 | 26.4 | 57.1 | 29.8 | 4.62 | 17.1 | 45 | 412 | 5 | TRUE |
| 46 | 8.2 | 26.3 | 62.2 | 31.3 | 4.22 | 18.6 | 14 | 463 | 3 | TRUE |
| 48 | 8.1 | 26.7 | 62.1 | 30.7 | 4.31 | 19 | 11 | 449 | 3 | TRUE |
| 53 | 9.8 | 31.8 | 57.3 | 30.8 | 5.54 | 22.9 | 11 | 405 | 12 | TRUE |
| 53 | 9.6 | 29.8 | 65.4 | 32.1 | 4.55 | 18 | 34 | 432 | 4 | TRUE |
| 21 | 14.9 | 40.5 | 86.1 | 36.1 | 4.7 | 13.5 | 124 | 283 | 205 | FALSE |
| 24 | 15.3 | 43.2 | 82 | 35.5 | 5.2 | 3.5 | 88 | 288 | 240 | FALSE |
| 27 | 11.4 | 32.2 | 85.8 | 35.4 | 3.7 | 13.1 | 103 | 177 | 640 | FALSE |
| 28 | 13.8 | 38.2 | 82.6 | 36.2 | 4.6 | 13.1 | 61 | 281 | 48 | FALSE |
| 31 | 13.1 | 37 | 82.3 | 35.4 | 4.5 | 15.8 | 64 | 339 | 42 | FALSE |
| 32 | 13.2 | 38.3 | 79.9 | 34.6 | 4.8 | 15.2 | 106 | 196 | 464 | FALSE |
| 34 | 14.1 | 40.5 | 79 | 34.8 | 5.1 | 14.1 | 40 | 332 | 44 | FALSE |
| 35 | 15.3 | 43.8 | 88.8 | 34.9 | 4.9 | 12.6 | 74 | 192 | 77 | FALSE |
| 36 | 13.4 | 39.7 | 78.4 | 33.8 | 5 | 27.5 | 48 | 226 | 164 | FALSE |
| 36 | 12.8 | 36.2 | 89.7 | 35.2 | 4 | 14.2 | 60 | 328 | 48 | FALSE |
| 37 | 14.9 | 41.9 | 98.9 | 35.6 | 4.2 | 12.4 | 111 | 236 | 67 | FALSE |
| 38 | 12.8 | 35.7 | 83.8 | 36.2 | 4.26 | 25.2 | 122 | 182 | 165 | FALSE |
| 39 | 13.6 | 40.6 | 90 | 33.6 | 4.5 | 12.8 | 74 | 182 | 52 | FALSE |
| 40 | 13.4 | 38 | 83.3 | 35.2 | 4.5 | 27.9 | 113 | 161 | 214 | FALSE |
| 43 | 11.7 | 34.5 | 74.8 | 34.2 | 4.5 | 31.5 | 60 | 234 | 244 | FALSE |
| 44 | 13 | 37.2 | 81.5 | 34.9 | 4.5 | 18.2 | 102 | 203 | 157 | FALSE |
| 44 | 13.3 | 37.4 | 87.5 | 35.6 | 4.2 | 14 | 60 | 298 | 193 | FALSE |
| 45 | 15.7 | 45 | 87.3 | 34.5 | 5.1 | 128 | 60 | 280 | 121 | FALSE |
| 46 | 17.3 | 48.8 | 84.8 | 35.7 | 5.7 | 13.2 | 95 | 323 | 45 | FALSE |
| 46 | 15.6 | 45 | 82.2 | 34.6 | 5.4 | 13.2 | 115 | 293 | 67 | FALSE |
| 47 | 14.6 | 41.2 | 82.9 | 35.5 | 4.9 | 27.7 | 74 | 271 | 105 | FALSE |
| 48 | 15.8 | 45.1 | 86.3 | 35 | 5.2 | 14.1 | 68 | 223 | 79 | FALSE |
| 51 | 15.6 | 43.8 | 91.1 | 35.6 | 4.8 | 13.8 | 151 | 243 | 122 | FALSE |
| 52 | 12.8 | 36.5 | 85.2 | 34.9 | 4.2 | 15.2 | 70 | 230 | 148 | FALSE |
| 53 | 11.6 | 33.6 | 81.5 | 34.4 | 4.1 | 16 | 69 | 366 | 68 | FALSE |
| 53 | 12.3 | 37.1 | 64.9 | 33.2 | 5.7 | 13.9 | 90 | 265 | 130 | FALSE |
| 56 | 13.9 | 38.7 | 86.3 | 35.9 | 4.5 | 14.2 | 105 | 244 | 54 | FALSE |
| 60 | 13.4 | 39.2 | 86.8 | 34.2 | 4.5 | 13.3 | 63 | 304 | 49 | FALSE |
| 61 | 12.2 | 35.2 | 80.7 | 34.6 | 4.3 | 14.3 | 60 | 276 | 70 | FALSE |
| 64 | 13.5 | 38 | 89.6 | 35.6 | 4.2 | 13.1 | 135 | 211 | 59 | FALSE |
| 65 | 14.5 | 41.8 | 78.7 | 34.7 | 5.3 | 15.2 | 133 | 254 | 110 | FALSE |
| 72 | 14.8 | 42.7 | 87.1 | 34.7 | 4.9 | 12.6 | 117 | 388 | 74 | FALSE |
| 72 | 12.7 | 35 | 97.2 | 35.7 | 3.6 | 13.9 | 74 | 294 | 54 | FALSE |
| 59 | 15.2 | 42.6 | 85.9 | 35.6 | 4.9 | 13 | 95 | 199 | 118 | FALSE |
| 59 | 13.8 | 41.6 | 83.7 | 33.2 | 4.9 | 14.5 | 65 | 345 | 45 | FALSE |
| 59 | 13.1 | 38.4 | 84.6 | 34.2 | 4.5 | 13.8 | 113 | 247 | 107 | FALSE |
| 58 | 14.1 | 41.1 | 82.7 | 34.4 | 4.9 | 14.7 | 122 | 263 | 40 | FALSE |
| 58 | 12.8 | 36.5 | 92.7 | 35 | 3.9 | 15.4 | 54 | 257 | 70 | FALSE |
| 57 | 14.2 | 40.6 | 78 | 34.9 | 5.1 | 13.8 | 41 | 404 | 51 | FALSE |
| 57 | 14.9 | 43.6 | 85.7 | 34.2 | 5 | 13.2 | 87 | 252 | 170 | FALSE |
| 54 | 14.3 | 40.5 | 82.8 | 35.2 | 4.9 | 14 | 60 | 255 | 48 | FALSE |
| 51 | 11.1 | 32.2 | 81.4 | 34.2 | 3.9 | 15.4 | 93 | 296 | 89 | FALSE |
| 49 | 13.2 | 38.7 | 79.9 | 34.2 | 4.8 | 14 | 78 | 339 | 47 | FALSE |
| 47 | 12.2 | 36.1 | 83.8 | 33.9 | 4.3 | 13.4 | 76 | 270 | 68 | FALSE |
| 45 | 12.8 | 36.7 | 94.8 | 34.7 | 3.8 | 15.2 | 110 | 258 | 115 | FALSE |
| 45 | 13.1 | 38.7 | 80.4 | 33.9 | 4.8 | 13.2 | 64 | 213 | 109 | FALSE |
| 70 | 12.6 | 36.9 | 82.6 | 34 | 4.4 | 15 | 78 | 281 | 69 | FALSE |
| 70 | 12.6 | 37.1 | 94.8 | 33.8 | 3.9 | 15.2 | 88 | 171 | 180 | FALSE |
| 70 | 12.7 | 37.2 | 84.3 | 34.1 | 4.4 | 12.9 | 71 | 308 | 50 | FALSE |
| 69 | 12.4 | 36.9 | 92.9 | 33.7 | 3.9 | 15.6 | 52 | 244 | 185 | FALSE |
| 66 | 11.9 | 34.7 | 87.3 | 34.4 | 3.9 | 13.9 | 94 | 226 | 190 | FALSE |
| 65 | 12.3 | 35 | 81.4 | 25.1 | 4.2 | 14.2 | 72 | 245 | 201 | FALSE |
| 65 | 12.9 | 37.6 | 89.7 | 34.2 | 4.1 | 12.3 | 90 | 173 | 88 | FALSE |
| 64 | 11.9 | 34.7 | 84.2 | 34.2 | 4.1 | 15.1 | 83 | 250 | 140 | FALSE |
| 63 | 14.4 | 41.5 | 89.9 | 34.7 | 4.6 | 13 | 63 | 235 | 64 | FALSE |
| 61 | 11 | 32.6 | 34.9 | 33.8 | 3.8 | 28 | 83 | 208 | 240 | FALSE |
| 60 | 13 | 37.5 | 86.2 | 34.5 | 4.3 | 13.3 | 75 | 242 | 72 | FALSE |
| 59 | 14.6 | 43.5 | 83.3 | 33.6 | 5.1 | 14.3 | 156 | 272 | 67 | FALSE |
| 59 | 12.8 | 37 | 86.6 | 34.5 | 4.2 | 13.1 | 68 | 251 | 75 | FALSE |
| 59 | 13.2 | 39 | 81.2 | 33.8 | 4.8 | 14 | 87 | 302 | 40 | FALSE |
| 57 | 12.6 | 38.6 | 77.4 | 32.5 | 4.9 | 17.4 | 56 | 220 | 78 | FALSE |
| 56 | 12.4 | 35.6 | 96 | 34.9 | 3.7 | 12.9 | 90 | 169 | 96 | FALSE |
| 54 | 11.8 | 33.3 | 87 | 35 | 3.2 | 14 | 100 | 252 | 124 | FALSE |
| 53 | 14.1 | 41.7 | 87.4 | 33.9 | 4.7 | 13.3 | 142 | 244 | 47 | FALSE |
| 49 | 12 | 34.7 | 90 | 34.5 | 3.8 | 16.5 | 67 | 213 | 142 | FALSE |
| 46 | 13.7 | 40.2 | 86.2 | 34 | 4.6 | 13.4 | 97 | 236 | 48 | FALSE |
| 45 | 14.3 | 42.1 | 86.7 | 34 | 4.8 | 16.4 | 92 | 360 | 159 | FALSE |
| 44 | 12.8 | 38.2 | 80.8 | 33.5 | 4.7 | 17 | 93 | 334 | 240 | FALSE |
| 44 | 13.1 | 39.1 | 80.3 | 33.4 | 4.8 | 15.7 | 118 | 158 | 277 | FALSE |
| 40 | 13.7 | 39.1 | 90.5 | 35 | 4.3 | 12.9 | 70 | 262 | 102 | FALSE |
| 32 | 12.6 | 37 | 87.8 | 34.1 | 4.2 | 12.8 | 98 | 218 | 135 | FALSE |
| 28 | 15.7 | 45.9 | 86.9 | 34.2 | 5.2 | 13 | 128 | 277 | 45 | FALSE |
| 27 | 15 | 43.2 | 86.2 | 34.8 | 5 | 14.7 | 74 | 215 | 92 | FALSE |
| 88 | 13.2 | 38.9 | 92.4 | 33.9 | 4.2 | 14.4 | 65 | 238 | 58 | FALSE |
| 81 | 12.6 | 36.6 | 94.8 | 34.4 | 3.8 | 13.5 | 79 | 267 | 54 | FALSE |
| 79 | 15.1 | 45 | 88.2 | 33.6 | 5.1 | 14.4 | 136 | 199 | 104 | FALSE |
| 75 | 13.9 | 39.9 | 84.5 | 34.7 | 4.7 | 13.8 | 82 | 281 | 76 | FALSE |
| 74 | 12.7 | 38.5 | 82.6 | 32.9 | 4.6 | 21.7 | 53 | 247 | 294 | FALSE |
| 73 | 12 | 35.4 | 81.1 | 33.9 | 4.3 | 13.8 | 69 | 257 | 50 | FALSE |
| 60 | 12.7 | 35.9 | 90.2 | 32.4 | 3.7 | 13.4 | 71 | 229 | 192 | FALSE |
| 68 | 11.8 | 34.8 | 80.1 | 33.8 | 4.3 | 14.9 | 65 | 277 | 75 | FALSE |
| 67 | 12.1 | 34.7 | 78.3 | 34.9 | 4.4 | 14.4 | 73 | 272 | 44 | FALSE |
| 64 | 12.9 | 37.6 | 86.6 | 34.3 | 4.3 | 13.6 | 83 | 289 | 82 | FALSE |
| 63 | 12.6 | 37.3 | 83.2 | 33.8 | 4.4 | 15.6 | 64 | 320 | 238 | FALSE |
| 58 | 14.4 | 40.1 | 87.3 | 35.8 | 4.6 | 12 | 99 | 217 | 52 | FALSE |
| 57 | 14.7 | 43.4 | 83.4 | 33.9 | 5.2 | 16.3 | 65 | 273 | 65 | FALSE |
| 57 | 13.8 | 41.1 | 79 | 33.6 | 5.2 | 13.8 | 93 | 215 | 106 | FALSE |
| 56 | 13 | 38.1 | 83.7 | 34.2 | 4.5 | 13.4 | 87 | 281 | 45 | FALSE |
| 54 | 13.3 | 38.6 | 82.3 | 34.3 | 4.6 | 14.3 | 67 | 310 | 46 | FALSE |
| 53 | 13.2 | 38 | 81.4 | 34.7 | 4.6 | 14.1 | 80 | 259 | 83 | FALSE |
| 51 | 13.4 | 38.7 | 85.3 | 34.8 | 4.5 | 13.3 | 93 | 265 | 67 | FALSE |
| 48 | 14.4 | 41.3 | 82.9 | 34.5 | 4.9 | 13.9 | 67 | 313 | 90 | FALSE |
| 47 | 14.6 | 41.9 | 86.4 | 34.8 | 4.8 | 12.2 | 70 | 352 | 41 | FALSE |
| 47 | 12.8 | 36.7 | 80 | 34.8 | 4.7 | 16 | 102 | 160 | 117 | FALSE |
| 35 | 13.5 | 39.1 | 83.7 | 34.5 | 4.6 | 16 | 144 | 176 | 65 | FALSE |
| 42 | 13.2 | 39.3 | 79.6 | 33.6 | 4.9 | 18.5 | 67 | 213 | 210 | FALSE |
| 42 | 11.8 | 35.3 | 71.7 | 33.5 | 4.9 | 27 | 60 | 205 | 160 | FALSE |
| 45 | 12.7 | 37.1 | 85.3 | 34.3 | 4.3 | 13 | 160 | 184 | 40 | FALSE |
| 45 | 13.7 | 41.2 | 77.9 | 33.2 | 5.2 | 24 | 112 | 203 | 113 | FALSE |
| 45 | 13.3 | 38.8 | 92.1 | 34.4 | 4.2 | 13 | 110 | 211 | 40 | FALSE |
| 51 | 14.7 | 42.5 | 88.2 | 34.4 | 4.7 | 13 | 107 | 216 | 129 | FALSE |
| 52 | 13.6 | 40.4 | 84.1 | 33.7 | 4.8 | 17 | 60 | 235 | 121 | FALSE |
| 82 | 11.8 | 34.1 | 83.4 | 34.7 | 4 | 16.7 | 67 | 269 | 47 | FALSE |
| 80 | 12.9 | 37.7 | 89 | 34.2 | 4.2 | 14 | 71 | 215 | 98 | FALSE |
| 79 | 11.2 | 34.7 | 81.7 | 32.2 | 4.2 | 16.3 | 93 | 257 | 46 | FALSE |
| 72 | 13 | 38.5 | 84.4 | 33.7 | 4.1 | 14.6 | 57 | 226 | 108 | FALSE |
| 68 | 11.5 | 34.2 | 87.3 | 33.6 | 3.9 | 16 | 80 | 189 | 51 | FALSE |
| 67 | 11 | 32 | 94.5 | 35.1 | 3.2 | 14 | 89 | 263 | 40 | FALSE |
| 65 | 12.3 | 36 | 91.9 | 34.8 | 3.8 | 15.3 | 86 | 246 | 94 | FALSE |
| 61 | 15.8 | 45.1 | 93 | 35 | 4.8 | 13 | 125 | 138 | 66 | FALSE |
| 58 | 14.6 | 41.9 | 83.4 | 34.8 | 5 | 13.2 | 104 | 188 | 34 | FALSE |
| 56 | 14.8 | 42.8 | 80.7 | 34.5 | 4.3 | 13.3 | 64 | 292 | 64 | FALSE |
| 54 | 15.8 | 45.8 | 87.8 | 34.6 | 5.2 | 13.2 | 71 | 273 | 115 | FALSE |
| 53 | 14 | 40 | 87.2 | 34.8 | 4.5 | 15.8 | 64 | 236 | 187 | FALSE |
| 53 | 14.5 | 42.9 | 85.5 | 33.9 | 5 | 14.2 | 68 | 274 | 28 | FALSE |
| 52 | 12.8 | 36.1 | 87.7 | 35.5 | 4.1 | 12.3 | 105 | 235 | 37 | FALSE |
| 51 | 13 | 38 | 89.1 | 34.2 | 4.2 | 12 | 97 | 220 | 219 | FALSE |
| 50 | 14.2 | 41 | 86.5 | 34.2 | 4.7 | 14.1 | 70 | 256 | 64 | FALSE |
| 50 | 13.9 | 40.5 | 87.5 | 34.3 | 4.6 | 13.1 | 111 | 299 | 42 | FALSE |
| 49 | 14.2 | 41.8 | 80.5 | 34 | 5.1 | 28 | 108 | 169 | 125 | FALSE |
| 48 | 13.6 | 39.6 | 82.3 | 34.5 | 4.8 | 14 | 57 | 280 | 90 | FALSE |
| 43 | 13.2 | 39.5 | 79.1 | 33.4 | 4.9 | 20 | 126 | 205 | 72 | FALSE |
| 40 | 14.5 | 41.9 | 84 | 34.6 | 4.9 | 15.7 | 77 | 236 | 53 | FALSE |
| 38 | 11 | 32.7 | 89 | 33.6 | 3.6 | 16 | 61 | 378 | 56 | FALSE |
| 35 | 15.7 | 46 | 78 | 33 | 5.9 | 15 | 81 | 314 | 97 | FALSE |
| 34 | 12.8 | 37.5 | 82 | 34.2 | 4.5 | 21.2 | 115 | 135 | 199 | FALSE |
| 23 | 15.6 | 47.7 | 86.2 | 32.8 | 5.1 | 13 | 124 | 228 | 184 | FALSE |
| 40 | 12.6 | 37.2 | 84.3 | 33.7 | 4.4 | 15.8 | 77 | 274 | 133 | FALSE |
| 41 | 16.2 | 47.1 | 87.3 | 34.2 | 5.4 | 12.5 | 74 | 349 | 288 | FALSE |
| 42 | 13.5 | 39.5 | 82.5 | 34.1 | 4.7 | 13.3 | 68 | 241 | 45 | FALSE |
| 49 | 14.8 | 43.5 | 80.9 | 34.1 | 5.3 | 13.3 | 104 | 244 | 134 | FALSE |
| 50 | 15 | 43.5 | 88.7 | 34.4 | 4.9 | 13.4 | 84 | 241 | 59 | FALSE |
| 53 | 12.1 | 34.2 | 86.4 | 35.2 | 3.9 | 13.2 | 72 | 228 | 104 | FALSE |
| 55 | 13.3 | 40.1 | 77.5 | 33.1 | 5.1 | 19.8 | 67 | 201 | 220 | FALSE |
| 55 | 13.4 | 39.9 | 80 | 33.5 | 5 | 19.7 | 89 | 276 | 44 | FALSE |
| 56 | 13.1 | 37.6 | 87.2 | 34.7 | 4.3 | 13.9 | 78 | 266 | 46 | FALSE |
| 56 | 15.4 | 46.3 | 85.3 | 34.2 | 5.1 | 14.6 | 54 | 293 | 91 | FALSE |
| 57 | 15.2 | 44.1 | 83.3 | 34.4 | 5.3 | 16.3 | 71 | 291 | 171 | FALSE |
| 59 | 11.4 | 32.9 | 84.2 | 33.5 | 3.9 | 17.7 | 64 | 254 | 230 | FALSE |
| 74 | 11.3 | 33.1 | 89.1 | 34.2 | 3.7 | 15.1 | 99 | 222 | 81 | FALSE |
| 70 | 12.8 | 37.7 | 84 | 33.7 | 4.5 | 13.7 | 57 | 310 | 45 | FALSE |
| 52 | 13.1 | 38.2 | 86.6 | 34.2 | 4.4 | 13 | 93 | 290 | 46 | FALSE |
| 43 | 11.3 | 33.2 | 90 | 34 | 3.6 | 15 | 96 | 284 | 132 | FALSE |
| 21 | 14.8 | 41.8 | 80 | 35.2 | 5.2 | 16.6 | 80 | 237 | 90 | FALSE |
| 89 | 11.2 | 34.7 | 86.6 | 32.4 | 4 | 15.7 | 61 | 179 | 128 | FALSE |
| 86 | 11.4 | 33.4 | 80 | 34.1 | 3.1 | 15.1 | 122 | 223 | 174 | FALSE |
| 82 | 11.5 | 33.2 | 95 | 34.7 | 3.4 | 13.9 | 217 | 298 | 75 | FALSE |
| 80 | 11.9 | 35.2 | 83.6 | 33.9 | 4.2 | 15.2 | 68 | 290 | 50 | FALSE |
| 75 | 15 | 43.7 | 89.6 | 34.2 | 4.8 | 13.7 | 92 | 262 | 49 | FALSE |
| 75 | 13.7 | 40.7 | 88.4 | 34.1 | 4.2 | 13 | 157 | 240 | 124 | FALSE |
| 71 | 12.5 | 36.2 | 99.7 | 35.5 | 3.6 | 15.8 | 107 | 211 | 110 | FALSE |
| 71 | 12.1 | 34 | 87.7 | 35.1 | 3.8 | 14.4 | 85 | 338 | 47 | FALSE |
| 70 | 13.5 | 38.2 | 90 | 35.2 | 4.2 | 13.9 | 70 | 281 | 51 | FALSE |
| 70 | 13.9 | 39.7 | 84.8 | 35.1 | 4.6 | 15.8 | 80 | 156 | 124 | FALSE |
| 69 | 12.3 | 34.9 | 88.4 | 34.7 | 3.8 | 14.7 | 63 | 257 | 63 | FALSE |
| 67 | 11.3 | 34.5 | 85 | 34 | 3.9 | 14 | 74 | 238 | 120 | FALSE |
| 67 | 13.5 | 37 | 90 | 36 | 4.1 | 13 | 70 | 245 | 173 | FALSE |
