# Supplementary material for: A Novel Expert System for Diagnosis of Iron Deficiency Anemia
Source: Comput Math Methods Med. 2022 Oct 14;2022:7352096. doi: 10.1155/2022/7352096 (PMC9586777; doi:10.1155/2022/7352096)
Supplement: Supplementary Materials — Dataset and R codes used in the study are available as supplementary files online. [file 7352096.f1.zip › R codes.docx]

*##----------------------------------------------------------------*

*## SMOTE -*

*##----------------------------------------------------------------*

SMOTE <- function(x_train, y_train, k_neighbour = 5, imb_rate = NULL, class_neg = NULL, class_pos = NULL){

*#n_train <- nrow(x_train)*

p <- ncol(x_train)

**if** (**is**.null(class_neg) & **is**.null(class_pos)) {

class_names <- unique(y_train)

class_neg <- names(which.max(table(y_train)))

class_pos <- **as**.character(class_names[class_names != class_neg])

} **else** {

class_names <- c(class_neg, class_pos)

}

x_train_neg <- **as**.matrix(x_train[y_train == class_neg,])

x_train_pos <- **as**.matrix(x_train[y_train == class_pos,])

n_neg <- nrow(x_train_neg)

n_pos <- nrow(x_train_pos)

k_neighbour <- pmin(n_pos, k_neighbour)

**if** (**is**.null(imb_rate)) {

imb_rate <- n_neg/n_pos

} **else** {

imb_rate <- imb_rate

}

class_diff <- round(n_pos*(imb_rate - 1))

m_knn <- RANN::nn2(data = (x_train_pos), query = (x_train_pos), k = k_neighbour)

nn_pos <- m_knn$nn.idx

n_syn_for_each <- rep(floor(imb_rate - 1), n_pos)

missing <- class_diff - sum(n_syn_for_each)

selected <- sample(1:n_pos, missing)

n_syn_for_each[selected] <- n_syn_for_each[selected] + 1

n_syn <- sum(n_syn_for_each)

x_syn <- NULL

**for** (i **in** 1:n_pos) {

nn <- x_train_pos[nn_pos[i,],]

nn <- nn[sample(1:k_neighbour, size = n_syn_for_each[i], replace = TRUE),]

xx_pos <- matrix(unlist(x_train_pos[i,]), nrow = n_syn_for_each[i], ncol = p, byrow = TRUE)

rand <- runif(n_syn_for_each[i], 0, 1)

x_syn <- rbind(x_syn, xx_pos + (nn - xx_pos)*rand)

}

colnames(x_syn) <- colnames(x_train)

y_new <- c(

rep(**as**.character(class_pos), n_pos),

rep(**as**.character(class_pos), n_syn),

rep(**as**.character(class_neg), n_neg)

)

y_new <- factor(y_new, levels = class_names, labels = class_names)

x_new <- rbind(x_train_pos,

x_syn,

x_train_neg)

**return**(list(x_new = x_new,

y_new = y_new))

}

*##----------------------------------------------------------------*

*##----------------------------------------------------------------*

*## packages -*

*##----------------------------------------------------------------*

library(caret)

library(rbooster)

library(ggplot2)

library(GGally)

library(mvoutlier)

library(Boruta)

library(NoiseFiltersR)

library(patchwork)

*##----------------------------------------------------------------*

set.seed(1)

*##----------------------------------------------------------------*

*## data preparation -*

*##----------------------------------------------------------------*

data <- read.csv(file = "dataset.csv", sep = ";")

colnames(data) <- c("Age",

"Hb",

"Hct",

"MCV",

"MCHC",

"RBC",

"RDW",

"Fe",

"UIBC",

"FERR",

"DD")

data$DD <- **as**.factor(data$DD)

p <- ncol(data) - 1

n <- nrow(data)

x <- data[,1:p]

y <- data[,p+1]

*##----------------------------------------------------------------*

*##---------------------------------------------------------------*

*## outlier removal -*

*##---------------------------------------------------------------*

x_clean_1 <- x

y_clean_1 <- y

repeat {

outliers_1 <- c()

**for** (i **in** 1:p) {

oo <- which(abs(scale(x_clean_1[,i])) > 3)

outliers_1 <- c(outliers_1,oo)

}

outliers_1 <- **as**.numeric(names(table(outliers_1)))

**if** (length(outliers_1) == 0) {

**break**

}

x_clean_1 <- x_clean_1[-outliers_1,]

y_clean_1 <- y_clean_1[-outliers_1]

}

x_clean_2 <- x

y_clean_2 <- y

out_score_RDOS <- DDoutlier::RDOS(x_clean_2)

repeat {

outliers_2 <- which(abs(scale(out_score_RDOS)) > 3)

**print**(outliers_2)

**if** (length(outliers_2) == 0) {

**break**

}

x_clean_2 <- x_clean_2[-outliers_2,]

y_clean_2 <- y_clean_2[-outliers_2]

out_score_RDOS <- out_score_RDOS[-outliers_2]

}

x_clean_3 <- x

y_clean_3 <- y

out_score_RDOS <- DDoutlier::NOF(x_clean_3)$NOF

repeat {

outliers_3 <- which(abs(scale(out_score_RDOS)) > 3)

**print**(outliers_3)

**if** (length(outliers_3) == 0) {

**break**

}

x_clean_3 <- x_clean_3[-outliers_3,]

y_clean_3 <- y_clean_3[-outliers_3]

out_score_RDOS <- out_score_RDOS[-outliers_3]

}

x_original <- x

y_original <- y

*##---------------------------------------------------------------*

*##---------------------------------------------------------------*

*## feature importance boruta -*

*##---------------------------------------------------------------*

m_fs_original <- Boruta(x_original, y_original, maxRuns = 500)

m_fs_clean_1 <- Boruta(x_clean_1, y_clean_1, maxRuns = 500)

m_fs_clean_2 <- Boruta(x_clean_2, y_clean_2, maxRuns = 1500)

m_fs_clean_3 <- Boruta(x_clean_3, y_clean_3, maxRuns = 500)

tbl_boruta_selected_features <- data.frame(original = m_fs_original$finalDecision,

Z = m_fs_clean_1$finalDecision,

RDOS = m_fs_clean_2$finalDecision,

NOF = m_fs_clean_3$finalDecision)

write.csv(x = tbl_boruta_selected_features, file = "boruta selected features.csv")

shadow_variables <- c("shadowMax",

"shadowMean",

"shadowMin")

name_order_original <- colnames(m_fs_original$ImpHistory)[sort.int(apply(m_fs_original$ImpHistory, 2, mean), index.**return** = TRUE)$ix]

tbl_boruta_fs_original <- data.frame(

Feature = factor(colnames(m_fs_original$ImpHistory), levels = name_order_original),

mean = apply(m_fs_original$ImpHistory, 2, mean),

se = apply(m_fs_original$ImpHistory, 2, function(m) sd(m)/sqrt(length(m))),

group = **as**.factor(ifelse(colnames(m_fs_original$ImpHistory) %in% shadow_variables, "shadow", "not shadow"))

)

nn_original <- nrow(m_fs_original$ImpHistory)

plt_boruta_original <- ggplot() +

geom_bar(mapping = aes(y = tbl_boruta_fs_original$Feature,

x = tbl_boruta_fs_original$mean,

fill = tbl_boruta_fs_original$group),

stat = "identity", show.legend = FALSE) +

geom_errorbar(mapping = aes(y = tbl_boruta_fs_original$Feature,

x = tbl_boruta_fs_original$mean,

xmin = tbl_boruta_fs_original$mean - qt(0.975, nn_original)*tbl_boruta_fs_original$se,

xmax = tbl_boruta_fs_original$mean + qt(0.975, nn_original)*tbl_boruta_fs_original$se),

width = 0.5) +

ggsci::scale_fill_d3() +

theme_bw() +

scale_x_continuous("Importance") +

scale_y_discrete("Feature") +

labs(title = "Original data") +

theme(plot.title = element_text(hjust = 0.5))

name_order_clean_1 <- colnames(m_fs_clean_1$ImpHistory)[sort.int(apply(m_fs_clean_1$ImpHistory, 2, mean), index.**return** = TRUE)$ix]

tbl_boruta_fs_clean_1 <- data.frame(

Feature = factor(colnames(m_fs_clean_1$ImpHistory), levels = name_order_clean_1),

mean = apply(m_fs_clean_1$ImpHistory, 2, mean),

se = apply(m_fs_clean_1$ImpHistory, 2, function(m) sd(m)/sqrt(length(m))),

group = **as**.factor(ifelse(colnames(m_fs_clean_1$ImpHistory) %in% shadow_variables, "shadow", "not shadow"))

)

nn_clean_1 <- nrow(m_fs_clean_1$ImpHistory)

plt_boruta_clean_1 <- ggplot() +

geom_bar(mapping = aes(y = tbl_boruta_fs_clean_1$Feature,

x = tbl_boruta_fs_clean_1$mean,

fill = tbl_boruta_fs_clean_1$group),

stat = "identity", show.legend = FALSE) +

geom_errorbar(mapping = aes(y = tbl_boruta_fs_clean_1$Feature,

x = tbl_boruta_fs_clean_1$mean,

xmin = tbl_boruta_fs_clean_1$mean - qt(0.975, nn_clean_1)*tbl_boruta_fs_clean_1$se,

xmax = tbl_boruta_fs_clean_1$mean + qt(0.975, nn_clean_1)*tbl_boruta_fs_clean_1$se),

width = 0.5) +

ggsci::scale_fill_d3() +

theme_bw() +

scale_x_continuous("Importance") +

scale_y_discrete("Feature") +

labs(title = "Z") +

theme(plot.title = element_text(hjust = 0.5))

name_order_clean_2 <- colnames(m_fs_clean_2$ImpHistory)[sort.int(apply(m_fs_clean_2$ImpHistory, 2, mean), index.**return** = TRUE)$ix]

tbl_boruta_fs_clean_2 <- data.frame(

Feature = factor(colnames(m_fs_clean_2$ImpHistory), levels = name_order_clean_2),

mean = apply(m_fs_clean_2$ImpHistory, 2, mean),

se = apply(m_fs_clean_2$ImpHistory, 2, function(m) sd(m)/sqrt(length(m))),

group = **as**.factor(ifelse(colnames(m_fs_clean_2$ImpHistory) %in% shadow_variables, "shadow", "not shadow"))

)

nn_clean_2 <- nrow(m_fs_clean_2$ImpHistory)

plt_boruta_clean_2 <- ggplot() +

geom_bar(mapping = aes(y = tbl_boruta_fs_clean_2$Feature,

x = tbl_boruta_fs_clean_2$mean,

fill = tbl_boruta_fs_clean_2$group),

stat = "identity", show.legend = FALSE) +

geom_errorbar(mapping = aes(y = tbl_boruta_fs_clean_2$Feature,

x = tbl_boruta_fs_clean_2$mean,

xmin = tbl_boruta_fs_clean_2$mean - qt(0.975, nn_clean_2)*tbl_boruta_fs_clean_2$se,

xmax = tbl_boruta_fs_clean_2$mean + qt(0.975, nn_clean_2)*tbl_boruta_fs_clean_2$se),

width = 0.5) +

ggsci::scale_fill_d3() +

theme_bw() +

scale_x_continuous("Importance") +

scale_y_discrete("Feature") +

labs(title = "RDOS") +

theme(plot.title = element_text(hjust = 0.5))

name_order_clean_3 <- colnames(m_fs_clean_3$ImpHistory)[sort.int(apply(m_fs_clean_3$ImpHistory, 2, mean), index.**return** = TRUE)$ix]

tbl_boruta_fs_clean_3 <- data.frame(

Feature = factor(colnames(m_fs_clean_3$ImpHistory), levels = name_order_clean_3),

mean = apply(m_fs_clean_3$ImpHistory, 2, mean),

se = apply(m_fs_clean_3$ImpHistory, 2, function(m) sd(m)/sqrt(length(m))),

group = **as**.factor(ifelse(colnames(m_fs_clean_3$ImpHistory) %in% shadow_variables, "shadow", "not shadow"))

)

nn_clean_3 <- nrow(m_fs_clean_3$ImpHistory)

plt_boruta_clean_3 <- ggplot() +

geom_bar(mapping = aes(y = tbl_boruta_fs_clean_3$Feature,

x = tbl_boruta_fs_clean_3$mean,

fill = tbl_boruta_fs_clean_3$group),

stat = "identity", show.legend = FALSE) +

geom_errorbar(mapping = aes(y = tbl_boruta_fs_clean_3$Feature,

x = tbl_boruta_fs_clean_3$mean,

xmin = tbl_boruta_fs_clean_3$mean - qt(0.975, nn_clean_3)*tbl_boruta_fs_clean_3$se,

xmax = tbl_boruta_fs_clean_3$mean + qt(0.975, nn_clean_3)*tbl_boruta_fs_clean_3$se),

width = 0.5) +

ggsci::scale_fill_d3() +

theme_bw() +

scale_x_continuous("Importance") +

scale_y_discrete("Feature") +

labs(title = "NOF") +

theme(plot.title = element_text(hjust = 0.5))

plt_all <- plt_boruta_original + plt_boruta_clean_1 + plt_boruta_clean_2 + plt_boruta_clean_3

ggsave(filename = "plt boruta all.png",

plot = plt_all,

device = "png",

width = 12,

height = 6,

dpi = 600)

*##--------------------------------------------------------------*

*##----------------------------------------------------------------*

*## Data summary -*

*##----------------------------------------------------------------*

tbl_summary_x <- rbind(

colMeans(x_original),

apply(x_original, 2, sd),

apply(x_original, 2, range),

colMeans(x_clean_1),

apply(x_clean_1, 2, sd),

apply(x_clean_1, 2, range),

colMeans(x_clean_2),

apply(x_clean_2, 2, sd),

apply(x_clean_2, 2, range),

colMeans(x_clean_3),

apply(x_clean_3, 2, sd),

apply(x_clean_3, 2, range)

)

tbl_summary_x <- t(tbl_summary_x)

colnames(tbl_summary_x) <- paste(rep(c("no outlier removal", "z", "RDOS", "NOF"), each = 4),

rep(c("mean", "sd", "min", "max"), 4))

write.csv(x = tbl_summary_x, file = "data summary x.csv")

tbl_summary_y <- rbind(

table(y_original),

table(y_clean_1),

table(y_clean_2),

table(y_clean_3)

)

tbl_summary_y <- **as**.data.frame(tbl_summary_y)

tbl_summary_y$imbalance <- tbl_summary_y[,1]/tbl_summary_y[,2]

rownames(tbl_summary_y) <- c("Original",

"Z",

"RDOS",

"NOF")

write.csv(x = tbl_summary_y, file = "data summary y.csv")

tbl_asd <- data.frame(E = abs(tbl_summary_y[1,1] - tbl_summary_y[,1]),

H = abs(tbl_summary_y[1,2] - tbl_summary_y[,2]))

tbl_asd <- cbind(tbl_asd, rowSums(tbl_asd))

*##----------------------------------------------------------------*

*##----------------------------------------------------------------*

*## pairs plot -*

*##----------------------------------------------------------------*

pairs_plot_original <- ggpairs(data = data.frame(x_original, DD = y_original), mapping = aes(color = DD),

upper = list(continuous = wrap("cor",

size = 2),

combo = wrap("box_no_facet",

size = 0.1,

outlier.size = 0.1),

discrete = "count",

na = "na"),

lower = list(continuous = wrap("points",

size = 0.1),

combo = "facethist",

combo = wrap("facetbar",

size = 0.1),

na = "na"),

diag = list(continuous = wrap("densityDiag",

size = 0.1))) +

ggsci::scale_color_d3() +

ggsci::scale_fill_d3() +

theme_bw(base_size = 7)

ggsave(filename = "pairs_plot_original.png",

plot = pairs_plot_original,

device = "png",

dpi = 300,

height = 5,

width = 10)

pairs_plot_clean_1 <- ggpairs(data = data.frame(x_clean_1, DD = y_clean_1), mapping = aes(color = DD),

upper = list(continuous = wrap("cor",

size = 2),

combo = wrap("box_no_facet",

size = 0.1,

outlier.size = 0.1),

discrete = "count",

na = "na"),

lower = list(continuous = wrap("points",

size = 0.1),

combo = "facethist",

combo = wrap("facetbar",

size = 0.1),

na = "na"),

diag = list(continuous = wrap("densityDiag",

size = 0.1))) +

ggsci::scale_color_d3() +

ggsci::scale_fill_d3() +

theme_bw(base_size = 7)

ggsave(filename = "pairs_plot_clean_z.png",

plot = pairs_plot_clean_1,

device = "png",

dpi = 300,

height = 5,

width = 10)

pairs_plot_clean_2 <- ggpairs(data = data.frame(x_clean_2, DD = y_clean_2), mapping = aes(color = DD),

upper = list(continuous = wrap("cor",

size = 2),

combo = wrap("box_no_facet",

size = 0.1,

outlier.size = 0.1),

discrete = "count",

na = "na"),

lower = list(continuous = wrap("points",

size = 0.1),

combo = "facethist",

combo = wrap("facetbar",

size = 0.1),

na = "na"),

diag = list(continuous = wrap("densityDiag",

size = 0.1))) +

ggsci::scale_color_d3() +

ggsci::scale_fill_d3() +

theme_bw(base_size = 7)

ggsave(filename = "pairs_plot_clean_RDOS.png",

plot = pairs_plot_clean_2,

device = "png",

dpi = 300,

height = 5,

width = 10)

pairs_plot_clean_3 <- ggpairs(data = data.frame(x_clean_3, DD = y_clean_3), mapping = aes(color = DD),

upper = list(continuous = wrap("cor",

size = 2),

combo = wrap("box_no_facet",

size = 0.1,

outlier.size = 0.1),

discrete = "count",

na = "na"),

lower = list(continuous = wrap("points",

size = 0.1),

combo = "facethist",

combo = wrap("facetbar",

size = 0.1),

na = "na"),

diag = list(continuous = wrap("densityDiag",

size = 0.1))) +

ggsci::scale_color_d3() +

ggsci::scale_fill_d3() +

theme_bw(base_size = 7)

ggsave(filename = "pairs_plot_clean_NOF.png",

plot = pairs_plot_clean_3,

device = "png",

dpi = 300,

height = 5,

width = 10)

*##----------------------------------------------------------------*

*##---------------------------------------------------------------*

*## metric function -*

*##---------------------------------------------------------------*

Summary_Fatih <- function (data, lev = NULL, model = NULL)

{

ACC <- mlr::measureACC(truth = data[,"obs"], response = data[,"pred"])

MCC <- mlr::measureMCC(truth = data[,"obs"], response = data[,"pred"], negative = lev[2], positive = lev[1])

AUC <- mlr::measureAUC(probabilities = data[,lev[1]], truth = data[,"obs"], negative = lev[2], positive = lev[1])

Spec <- mlr::measureTNR(truth = data[,"obs"], response = data[,"pred"], negative = lev[2])

Sens <- mlr::measureTPR(truth = data[,"obs"], response = data[,"pred"], positive = lev[1])

out <- c(ACC, MCC, AUC, Spec, Sens)

names(out) <- c("ACC", "MCC", "AUC", "Spec", "Sens")

out

}

*##---------------------------------------------------------------*

*##----------------------------------------------------------------*

*## Final datasets -*

*##----------------------------------------------------------------*

x_original_fs <- x_original[,m_fs_original$finalDecision == "Confirmed"]

x_clean_1_fs <- x_clean_1[,m_fs_clean_1$finalDecision == "Confirmed"]

x_clean_2_fs <- x_clean_2[,m_fs_clean_2$finalDecision == "Confirmed"]

x_clean_3_fs <- x_clean_3[,m_fs_clean_3$finalDecision == "Confirmed"]

n_original <- nrow(x_original)

p_original <- ncol(x_original)

n_clean_1 <- nrow(x_clean_1)

p_clean_1 <- ncol(x_clean_1)

n_clean_2 <- nrow(x_clean_2)

p_clean_2 <- ncol(x_clean_2)

n_clean_3 <- nrow(x_clean_3)

p_clean_3 <- ncol(x_clean_3)

x_list <- list(x_original,

x_clean_1,

x_clean_2,

x_clean_3)

y_list <- list(y_original,

y_clean_1,

y_clean_2,

y_clean_3)

names(x_list) <- c("Original",

"Z",

"RDOS",

"NOF")

names(y_list) <- c("Original",

"Z",

"RDOS",

"NOF")

*##----------------------------------------------------------------*

*##----------------------------------------------------------------*

*## TSNE plots -*

*##----------------------------------------------------------------*

tsne_plots <- list()

**for** (i **in** 1:length(x_list)) {

tsne_plots[[i]] <- local({

i <- i

m_tsne_variables <- Rtsne::Rtsne(x_list[[i]], pca = FALSE)$Y

m_nf <- NoiseFiltersR::EF(x = data.frame(m_tsne_variables, y_list[[i]]))

ggplot() +

geom_point(mapping = aes(x = m_tsne_variables[,1],

y = m_tsne_variables[,2],

color = y_list[[i]], alpha = 0.7),

show.legend = FALSE) +

ggsci::scale_color_d3() +

theme_bw() +

geom_point(mapping = aes(x = m_tsne_variables[m_nf$remIdx,1],

y = m_tsne_variables[m_nf$remIdx,2]),

show.legend = FALSE, size = 10, shape = 1, alpha = 0.5) +

ggsci::scale_fill_d3() +

scale_x_continuous(name = "Component 1", expand = c(0.2, 0.2)) +

scale_y_continuous(name = "Component 2", expand = c(0.2, 0.2)) +

labs(title = names(x_list)[i]) +

theme(plot.title = element_text(hjust = 0.5))

})

}

plt_tsne_all <- ggpubr::ggarrange(plotlist = tsne_plots, nrow = 1)

plt_tsne_all

ggsave(filename = "plt_tsne_all.png", plot = plt_tsne_all, device = "png", width = 13, height = 3)

*##----------------------------------------------------------------*

*##----------------------------------------------------------------*

*## preprocess functions -*

*##----------------------------------------------------------------*

noresampling <- list(

name = "noresampling",

func = function(x, y) {

list(x = **as**.matrix(x),

y = y)

},

first = TRUE

)

noise_filter <- list(

name = "noise_filter",

func = function(x, y) {

class_neg <- names(which.max(table(y)))

m_noise_filter <- **try**(NoiseFiltersR::EF(data.frame(**as**.data.frame(x),y), nfolds = 10), silent = TRUE)

**if** (**class**(m_noise_filter) == "try-error") {

m_noise_filter <- NoiseFiltersR::EF(data.frame(**as**.data.frame(x),y), nfolds = 10)

}

noises <- m_noise_filter$remIdx[which(y[m_noise_filter$remIdx] == class_neg)]

*# noises <- m_noise_filter$remIdx*

**if** (identical(noises, integer(0))) {

x <- x

y <- y

} **else** {

x <- x[-noises,]

y <- y[-noises]

}

list(x = **as**.matrix(x),

y = y)

},

first = TRUE

)

smote <- list(

name = "smote",

func = function(x, y) {

dat <- SMOTE(x_train = x, y_train = y)

list(x = **as**.matrix(dat$x_new),

y = dat$y_new)

},

first = TRUE

)

noise_filter_smote <- list(

name = "noise_filter_smote",

func = function(x, y) {

class_neg <- names(which.max(table(y)))

m_noise_filter <- **try**(NoiseFiltersR::EF(data.frame(**as**.data.frame(x),y), nfolds = 10), silent = TRUE)

**if** (**class**(m_noise_filter) == "try-error") {

m_noise_filter <- NoiseFiltersR::EF(data.frame(**as**.data.frame(x),y), nfolds = 10)

}

noises <- m_noise_filter$remIdx[which(y[m_noise_filter$remIdx] == class_neg)]

*#noises <- m_noise_filter$remIdx*

**if** (identical(noises, integer(0))) {

x <- x

y <- y

} **else** {

x <- x[-noises,]

y <- y[-noises]

}

dat <- SMOTE(x_train = x, y_train = y)

list(x = **as**.matrix(dat$x_new),

y = dat$y_new)

},

first = TRUE

)

pre_process_list <- list(noresampling,

noise_filter,

smote,

noise_filter_smote)

*##----------------------------------------------------------------*

*##----------------------------------------------------------------*

*## models -*

*##----------------------------------------------------------------*

models <- list()

**for** (i **in** 1:4) {

models[[i]] <- list()

}

names(models) <- c("Original",

"Z",

"RDOS",

"NOF")

**for** (i **in** 1:length(x_list)) {

rownames(x_list[[i]]) <- NULL

**for** (j **in** 1:length(pre_process_list)) {

m <- train(x = **as**.matrix(x_list[[i]]),

y = y_list[[i]],

method = "xgbLinear",

preProcess = c("scale", "center"),

tuneGrid = expand.grid(**lambda** = c(0, 0.25, 0.5, 0.75, 1),

alpha = c(0, 0.25, 0.5, 0.75, 1),

nrounds = c(50),

eta = c(0.01, 0.1, 0.25)),

trControl = trainControl(method = "repeatedcv",

number = 10,

repeats = 10,

classProbs = TRUE,

returnResamp = "all",

savePredictions = TRUE,

verboseIter = TRUE,

sampling = pre_process_list[[j]],

summaryFunction = Summary_Fatih),

metric = "MCC")

models[[i]][[j]] <- m

}

names(models[[i]]) <- c("No resampling", "EF", "SMOTE", "EF + SMOTE")

}

*##----------------------------------------------------------------*

*##----------------------------------------------------------------*

*## Final average results and sd -*

*##----------------------------------------------------------------*

**for** (i **in** 1:length(models)) {

pref_summary <- data.frame(matrix(NA, ncol = 10, nrow = 0))

best_tunes <- data.frame(matrix(NA, ncol = 4, nrow = 0))

**for** (j **in** 1:length(models[[i]])) {

results <- models[[i]][[j]]$results

best_tune <- models[[i]][[j]]$bestTune

ss <- which(apply(results[,1:4], 1, function(m) all(m == unlist(best_tune[,c(2,3,1,4)]))))

meanler <- results[ss,5:9]

sdler <- results[ss,10:14]

m_sd <- unlist(c(meanler, sdler))

pref_summary <- rbind(pref_summary, m_sd)

colnames(pref_summary) <- names(m_sd)

best_tunes <- rbind(best_tunes, best_tune)

colnames(best_tunes) <- names(best_tune)

}

rownames(pref_summary) <- names(models[[i]])

rownames(best_tunes) <- names(models[[i]])

write.csv(x = pref_summary,

file = paste("perf results", names(models)[i],".csv"))

write.csv(x = best_tunes,

file = paste("best tunes", names(models)[i],".csv"))

}

*##----------------------------------------------------------------*

*##---------------------------------------------------------------*

*## Var imp plots -*

*##---------------------------------------------------------------*

**for** (i **in** 1:length(models)) {

names(models[[i]]) <- c("No resampling", "EF", "SMOTE", "EF + SMOTE")

plts_varimp <- list()

tbls_varimp <- list()

**for** (j **in** 1:length(models[[i]])) {

vi <- varImp(models[[i]][[j]])

tbls_varimp[[j]] <- vi$importance

plts_varimp[[j]] <- local({

i <- i

j <- j

varimp <- varImp(models[[i]][[j]])

tbl <- data.frame(imp = unlist(varimp$importance),

variables = factor(rownames(varimp$importance),

levels = rev(rownames(varimp$importance))))

ggplot() +

geom_bar(mapping = aes(x = tbl$imp,

y = tbl$variables),

stat = "identity",

fill = ggsci::pal_d3()(1)) +

scale_y_discrete(name = "Features") +

scale_x_continuous(name = "Importance") +

theme_bw() +

labs(title = names(models[[1]])[j]) +

theme(plot.title = element_text(hjust = 0.5, size = 12))

})

}

plt_varimp <- plts_varimp[[1]] + plts_varimp[[2]] + plts_varimp[[3]] + plts_varimp[[4]] + plot_layout(nrow = 1)

ggsave(filename = paste("variable importance", names(models)[i], ".png"),

plot = plt_varimp,

device = "png",

width = 10,

height = 2.5,

dpi = 600)

tbl_varimp <- do.call(cbind, tbls_varimp)

write.csv(x = tbls_varimp,

file = paste("variable importance", names(models)[i], ".csv"))

}

*##---------------------------------------------------------------*

*##----------------------------------------------------------------*

*## ROC egrileri -*

*##----------------------------------------------------------------*

library(plotROC)

**for** (i **in** 1:length(models)) {

roc_plots <- list()

perf <- read.csv(file = paste("perf results", names(models)[i],".csv"))

**for** (j **in** 1:length(models[[i]])) {

roc_plots[[j]] <- local({

i <- i

j <- j

**print**(i)

**print**(j)

pred <- models[[i]][[j]]$pred

best_tune <- models[[i]][[j]]$bestTune

ss <- which(apply(pred[,c(6,7,8,9)], 1, function(m) all(m == unlist(best_tune[,c(2,3,1,4)]))))

dat_temp <- pred[ss,]

roc_plot_nores <- ggplot() +

geom_roc(aes(m = dat_temp$H,

d = dat_temp$obs),

size = 0.5, labels = FALSE) +

geom_abline(slope = 1,

intercept = 0,

alpha = 0.5,

linetype = 2,

color = "black") +

coord_equal() +

ggsci::scale_color_d3() +

labs(title = names(models[[i]])[j]) +

style_roc(xlab = "Specificity", ylab = "Sensitivity") +

theme(plot.title = element_text(hjust = 0.5, size = 12)) +

annotate(geom = "text",

x = 0.7,

y = 0.3,

label = paste0("AUC = ",

formatC(perf$AUC[j], digits = 4, format = "f")))

})

}

roc_plot <- ggpubr::ggarrange(roc_plots[[1]],

roc_plots[[2]],

roc_plots[[3]],

roc_plots[[4]],

nrow = 1,

ncol = 4,

common.legend = TRUE)

ggsave(filename = paste("roc", names(models)[i], ".png"),

plot = roc_plot,

device = "png",

width = 15,

height = 3.75,

dpi = 600)

}

*##----------------------------------------------------------------*

*##----------------------------------------------------------------*

*## Corr Plots -*

*##----------------------------------------------------------------*

cor_plots <- list()

**for** (i **in** 1:length(x_list)) {

cor_plots[[i]] <- local({

i <- i

cor_temp <- cor(x_list[[i]])

tbl_cor_temp <- reshape2::melt(cor_temp)

tbl_cor_temp$Var2 <- factor(tbl_cor_temp$Var2,

levels = rev(levels(tbl_cor_temp$Var2)))

tbl_cor_temp$text <- formatC(x = tbl_cor_temp$value,

digits = 3, format = "f")

ggplot() +

geom_tile(mapping = aes(x = tbl_cor_temp$Var1,

y = tbl_cor_temp$Var2,

fill = tbl_cor_temp$value)) +

scale_fill_gradient2(low = "#3B9AB2",

mid = "white",

high = "#F21A00",

midpoint = 0,

name = "Correlation", limits = c(-1,1)) +

scale_x_discrete(name = "") +

scale_y_discrete(name = "") +

labs(title = names(x_list)[i]) +

geom_text(mapping = aes(x = tbl_cor_temp$Var1,

y = tbl_cor_temp$Var2,

label = tbl_cor_temp$text)) +

theme_minimal() +

theme(plot.title = element_text(hjust = 0.5))

})

}

cor_plots_all <- ggpubr::ggarrange(plotlist = cor_plots, common.legend = TRUE, legend = "right")

ggsave(filename = "cor_plots_all.png", plot = cor_plots_all, device = "png", width = 17, height = 7, dpi = 600)

*##----------------------------------------------------------------*
